# PacC and pH–dependent transcriptome of the mycotrophic fungus *Trichoderma virens*

**DOI:** 10.1186/1471-2164-14-138

**Published:** 2013-02-28

**Authors:** Naomi Trushina, Michal Levin, Prasun K Mukherjee, Benjamin A Horwitz

**Affiliations:** 1 Department of Biology, Technion – Israel Institute of Technology, Haifa 32000, Israel; 2 Nuclear Agriculture and Biotechnology Division, Bhabha Atomic Research Centre, Trombay, Mumbai 440085, India; 3 Present address: Central Institute for Cotton Research, Nagpur 440010, India

## Abstract

**Background:**

In fungi, environmental pH is an important signal for development, and successful host colonization depends on homeostasis. Surprisingly, little is known regarding the role of pH in fungal-fungal interactions. Species of *Trichoderma* grow as soil saprobes but many are primarily mycotrophic, using other fungi as hosts. Therefore, *Trichoderma* spp*.* are studied for their potential in biocontrol of plant diseases. Particularly in alkaline soil, pH is a critical limiting factor for these biofungicides, whose optimal growth pH is 4–6. Gaining an understanding of pH adaptability is an important step in broadening the activity spectrum of these economically important fungi.

**Results:**

We studied the pH-responsive transcription factor PacC by gene knockout and by introduction of a constitutively active allele (*pacC*^*c*^). Δ*pacC* mutants exhibited reduced growth at alkaline pH, while *pacC*^*c*^ strains grew poorly at acidic pH. In plate confrontation assays Δ*pacC* mutants showed decreased ability to compete with the plant pathogens *Rhizoctonia solani* and *Sclerotium rolfsii*. The *pacC*^*c*^ strain exhibited an overgrowth of *R. solani* that was comparable to the wild type, but was unable to overgrow *S. rolfsii*. To identify genes whose expression is dependent on pH and *pacC*, we designed oligonucleotide microarrays from the transcript models of the *T. virens* genome, and compared the transcriptomes of wild type and mutant cultures exposed to high or low pH. Transcript levels from several functional classes were dependent on *pacC*, on pH, or on both. Furthermore, the expression of a set of *pacC*-dependent genes was increased in the constitutively-active *pacC*^*c*^ strain, and was pH-independent in some, but not all cases.

**Conclusions:**

PacC is important for biocontrol-related antagonism of other fungi by *T. virens*. As much as 5% of the transcriptome is pH-dependent, and of these genes, some 25% depend on *pacC*. Secondary metabolite biosynthesis and ion transport are among the relevant gene classes. We suggest that Δ*pacC* mutants may have lost their full biocontrol potential due to their inability to adapt to alkaline pH, to perceive ambient pH, or both. The results raise the novel possibility of genetically manipulating *Trichoderma* in order to improve adaptability and biocontrol at alkaline pH.

## Background

Fungi, whether they are saprophytes or pathogens, are able to detect and adapt to a wide range of environmental pH values. The ability of fungal cells to maintain homeostasis is exemplified in their capacity to grow in both acid and alkaline pH conditions. Furthermore, pH levels often serve as crucial external cues, providing information about the local environment. It is known that fungi respond to ambient pH levels via activation of a dedicated transcription factor, PacC [1-5]. This response is initiated by a signaling cascade which begins with a cell surface sensor, PalH, a protein with seven transmembrane helices. PalH probes the extracellular pH level. PalF, an arrestin-like protein which interacts with PalH, is ubiquitinated in response to alkaline pH [6]. The cascade leads to the activation of PacC by proteolytic cleavage and, finally, to transcriptional regulation [3,7-10].

Fungal pathogens of animals and plants need to sense and respond to local pH. In addition, several fungal pathogens of plants were shown to raise or lower the local pH during the establishment of the disease. It is also known that pH signals program gene expression in the pathogen [11-18]. For instance, *Colletotrichum gloeosporioides* attacks unripe avocado fruits, but these remain resistant to the fungus until the fruits ripen. During the ripening process, the pH of the avocado pericarp increases from pH 5.2 to pH 6.3, and following this increase in pH, the decay symptoms become evident. Furthermore, the pathogen itself contributes to alkalinization. Thus, the host pH can affect the progress of disease via the production of virulence factors [19]. There are several other examples which indicate the importance and relevance of PacC to this process: first, the *pacC* ortholog of *Colletotrichum acutatum* is essential for virulence on citrus, at the penetration stage [20]. Second, in the vascular wilt pathogen *Fusarium oxysporum*, constitutive-active *pacC* strains were found to be less virulent than wild type strains on the tomato host [5]. In contrast, *pacC* null mutants of the foliar pathogen *Sclerotinia sclerotiorum* were less virulent than wild type on *Arabidopsis* and tomato [21]. This contrasting regulation suggests that in different species, PacC may play different roles in determining virulence. Another interesting example is *Candida albicans*, a commensal that can become pathogenic in susceptible hosts. This organism colonizes the oral-pharyngeal, gastrointestinal, and urogenital tracts as a commensal. Following this colonization, *C. albicans* can become a pathogen, infecting these tissues. It may then enter the bloodstream and disseminate to almost any tissue in the body. The *C. albicans* sites of action are characterised by a diversity in pH levels, e.g., acidic pH levels in the gastrointestinal and vaginal tracts, and neutral-to-alkaline levels in the oral-pharyngeal tract and in the bloodstream. Thus, a successful response to extracellular pH is critical for virulence [22]. *Trichophyton rubrum,* a dermatophyte, responds to the pH level of human skin by expressing enzymes that have acid pH optima [23]. PacC has been shown to serve as a virulence factor for *Aspergillus nidulans* in a mouse model: null mutants showed reduced virulence, while a constitutively active *pacC* allele conferred hypervirulence [24]. The insect pathogen *Metarhizium anisopliae* uses pH as a signal to produce specific enzymes whose pH optima correspond to the ambient pH levels [25]. A wide variety of genes and activities are controlled by PacC, including xylanases [26], permeases [27], siderophores [28], antibiotic and toxin production [29], and involvement in pathogenic processes [4,21,24].

Fungi can also attack other fungi (either by direct mycoparasitism or, to give a more inclusive definition, mycotrophy [30]). Prominent examples of this are found among soil fungi of the genus *Trichoderma*, which are being applied worldwide as biocontrol agents against soil-borne and foliar plant diseases [31]. The biocontrol fungus antagonizes host fungi in a variety of ways: mycoparasitism, antibiotic production, induction of systemic resistance in the plant, competition for nutrients and space, modification of the local environment, and degradation of pathogen germination stimulants which are released by seeds. *Trichoderma* also increases the plant’s tolerance to stress by promoting growth and vigor [32-34]. Moreno-Mateos *et al.*[35] have shown that several *Trichoderma harzianum* genes which are thought to be involved in the antagonism of host fungi (chitinase, protease, glucose permease, and the cell wall protein qid74) are regulated by pH. In *T. harzianum*, PacC promotes the expression of chitinase and qid74, and represses protease and glucose permease. PacC also negatively regulates the production of inhibitory metabolites. Contrary to what might have been expected in view of the increased production of inhibitors, however, mycoparasitism is decreased in the null mutant [35]. In several *Trichoderma* species, conidiation depends on pH; the mode of regulation varies from one species or isolate to another [36]. Conidiation has previously been correlated with intracellular acidification [37], providing an explanation as to why the pH must be low in order for conidiation to occur on buffered media [36]. In this study, we examined an isolate of *Trichoderma virens* which is an aggressive mycoparasite on the sclerotia of *S. rolfsii* and on the hyphae and sclerotia of *Rhizoctonia solani*[38]. The genome sequence of this species has recently been made available [39,40], thus allowing a first complete analysis of pH- and PacC-regulation in a mycotroph at the level of the transcriptome.

## Results and discussion

### Construction of loss- and gain-of-function mutants in the pH-sensitive regulator *pacC*

#### T. virens pacC gene

Having initiated this study before the *T. virens* genome project, we used sequences from other filamentous ascomycetes to identify a *T. virens* ortholog of *pacC*. Degenerate primers for nested amplification (Additional file 1) were designed from the amino acid sequences of the regions most conserved in several *pacC* orthologs (*Aspergillus nidulans*, *Aspergillus oryzae* and *Fusarium oxysporum*). A product of ~400 bp was obtained and cloned, and four additional primers were designed for PCR-based genome walking. We obtained a genomic clone including ~2 kb of upstream and downstream sequences, sufficient for the design of gene replacement constructs. The gene contains a 2094 bp ORF with three predicted introns. The predicted protein product has a molecular mass of 67 kDa and contains 617 amino acid residues. When this sequence was compared to GenBank-deposited sequences from several organisms, the best BLAST similarity scores were obtained with the PacC proteins of the Ascomycete fungi *Fusarium oxysporum*, *Acremonium chrysogenum* and *Gibberella moniliformis* (62%, 64% and 67% similarity, respectively). Like *Trichoderma virens*, all of these species belong to the class Sordariomycetes. The consensus binding site for PacC identified from *Aspergillus nidulans*[41], GCCARG, is found 9 times within the 1 kb upstream of the predicted translation start of *T. virens pacC*. The strong, pH-independent expression of the truncated, active, *pacC*^*c*^ allele that we constructed suggests that PacC indeed regulates the expression of the gene that encodes it (see below). The sequence of the *T. virens* IMI 304061 *pacC* gene (this study, and genomic re-sequencing data, R. Normand, T. Katz-Ezov, N.T. and B.A.H., unpubl.) exhibits 24 SNPs relative to the corresponding gene model in *T. virens* Gv29.8 (the published reference strain [39]). These SNPs result in six predicted differences in protein sequence, four encoding amino acids with strongly similar properties to those of the sequence predicted from the reference genome, and two with weakly similar properties (Additional file 2). Thus, there are variations between two *Trichoderma virens* isolates in the amino acid sequence encoded by this well-conserved gene. It is unclear whether these SNPs have any functional significance.

#### pacC deletion mutants

To determine the function of *pacC*, deletion mutants (Δ*pacC*) were obtained by homologous integration resulting from double-crossover integration events. To this end, *T. virens* protoplasts were transformed with linearized plasmid DNA, using the polyethylene glycol-Ca^++^ method. Of about 100 transformants screened by PCR amplification with primers specific for homologous integration, we obtained two, Δ*pacC*1 and Δ*pacC*2, in which the *pacC* gene was replaced by the hygromycin resistance cassette. Transformants were purified by single-spore isolation, and homologous recombination was confirmed by PCR. The primer pairs, expected sizes, and PCR products obtained are provided in Additional file 3.

#### Construction of constitutively active pacC mutants

Constitutively active mutants (*pacC*^*c*^), are expected to bypass the need for the ambient pH signal, resulting in a phenotype that mimics growth under alkaline conditions [3,42-45]. Based on the known characterization of the proteolytic cascade activating *A. nidulans* PacC, we predicted that the truncation allele in *T. virens* that we designed (see below), would lead to a gain-of-function behavior, i.e. as a *bona fide pacC*^*c*^ allele. Indeed, transcriptional data, including data on the *pacC* gene itself, provide strong support for this assumption. To this end, we constructed a *pacC*^*c*^ line using the split-marker method ([46]; Additional file 4). To construct the *pacC*^*c*^ allele we designed a construct in which the C-terminal part of *pacC* (encoding amino acids 504–617, which are removed in processing upon activation in the cell) was replaced by the hygromycin resistance cassette. The construct also included flanking regions upstream and downstream of the gene, of ~1.1 kb each, to allow for double-crossover recombination. The transformants were isolated, purified, and confirmed by PCR amplification (Additional file 3).

#### Comparison of loss and gain-of function mutants to wt

**Figure 1:**
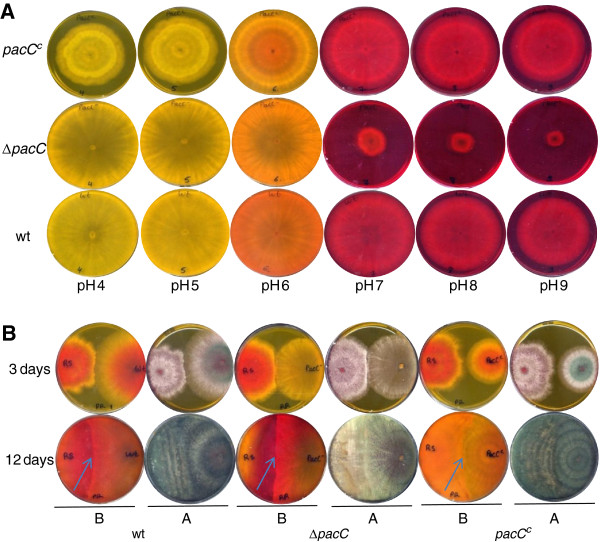
**A. Growth and colony phenotypes of loss and gain of function mutants in *pacC *on media of different pH.** Wild type, *pacC*^*c*^ and *ΔpacC* were assayed for ability to grow on PDA media buffered to different pH. For visualization of pH changes, the indicator Phenol Red was incorporated into the media, showing acidic (yellow) to basic (red) pH. Cultures were photographed after 5 days growth. **B**. Confrontation assays with *R. solani*. *T. virens* was coinoculated with *R. solani* by placing mycelial discs at the opposite sides of PDA plates. Overgrowth of *R. solani* by the different *T. virens* strains is evident from the production of green conidia; *R. solani* does not sporulate and appears white. The upper set of pictures was taken after 3 days, and the lower set after 12 days of growth. **A**, photos from above showing colony morphology and conidiation; **B**, photos from below showing pH gradients formed by the growing colonies.

**Figure 2:**
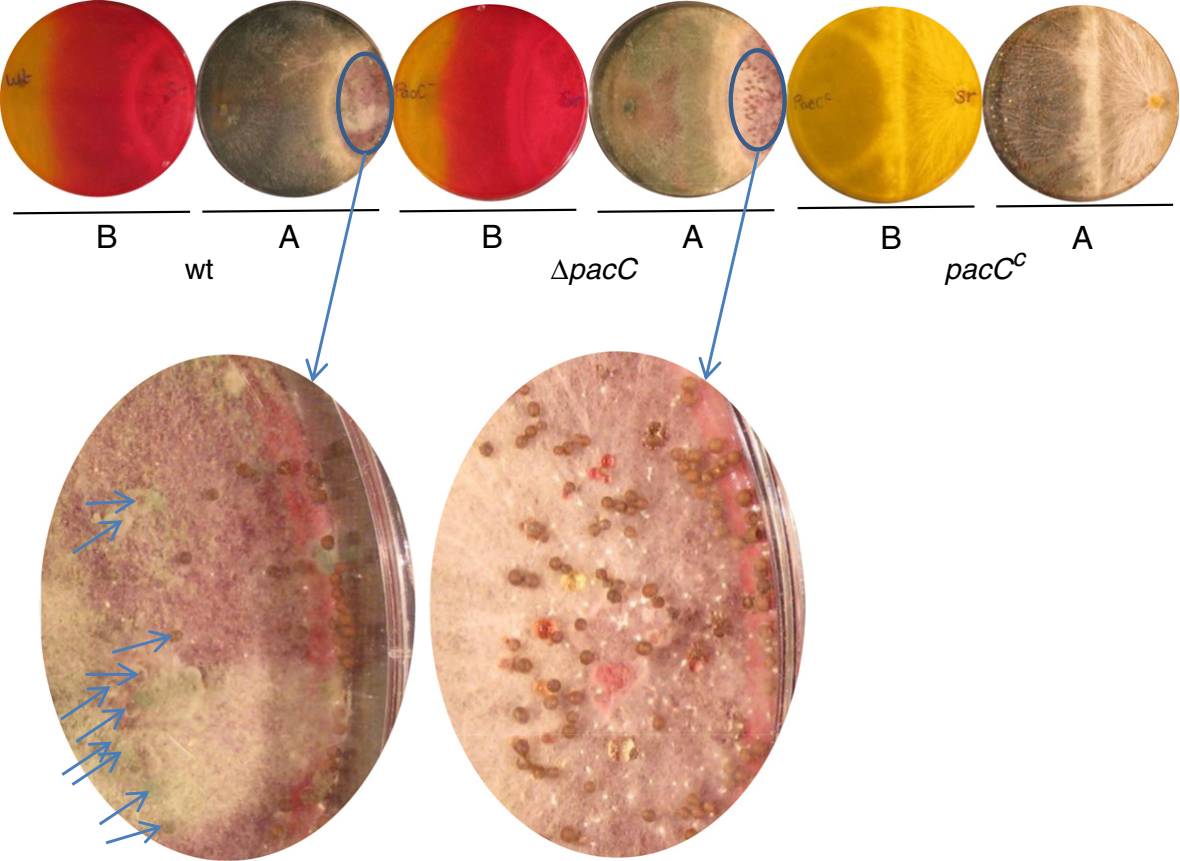
**Confrontation assay with *S. rolfsii*. ***T. virens* was coinoculated with *S. rolfsii* by placing mycelial discs at the opposite sides of PDA plates. Overgrowth of *S. rolfsii* by the different *T. virens* strains is evident from the production of green conidia; *S. rolfsii* does not sporulate and appears white. The picture was taken after 15 days of growth. Ellipses are magnified in the lower images in order to show sclerotia. Arrows in the wt image point to sclerotia which are nearly invisible because they are overgrown with sporulating *T. virens*. The indicator Phenol Red was incorporated into the media, showing acidic (yellow) to basic (red) pH. **A**, photos from above showing colony morphology and conidiation; **B**, photos from below showing pH gradients formed by the growing colonies.

During the first 24 h after inoculation, Δ*pacC* and wild-type strains exhibited nearly identical growth rates when inoculated on standard PDA (starting pH, 5.6) plates. After this initial time period the mutant displayed a slower growth rate than the wt strain, so that 48 h after the inoculation this resulted in a ~20% reduction of colony diameters of the Δ*pacC* mutant colonies grown in the light, and ~18% for diameters of colonies grown in the dark, when compared to wt colonies (Additional file 5). In addition, differences were apparent in the morphology of the colony surface. Colonies of the Δ*pacC* mutants conidiated less in the light (Additional file 6); in the dark, wild type colony surfaces were convex, whereas mutant colonies were flat. Wild type, *pacC*^*c*^ and Δ*pacC* strains were then assayed for their ability to grow on PDA media buffered to different pH values (buffer concentration 20 mM) (Figure 1A). The most striking phenotype of Δ*pacC* was observed at alkaline pH: there was a dramatic reduction in growth when the initial pH of the plates was 7 or higher (Figure 1A). For the *pacC*^c^ strain no such effect was evident, and its growth rate at alkaline pH was only slightly reduced in comparison to the wt (Figure 1A). Confrontation assays are often used as a measure of the ability of *Trichoderma* to compete with, to antagonize, and to overgrow a fungal host. We thus tested the wt, Δ*pacC* and *pacC*^c^ strains for their ability to overgrow and parasitize two plant pathogens, *Rhizoctonia solani* and *Sclerotium rolfsii*. Δ*pacC* overgrew *Rhizoctonia solani* more slowly than did the wt (Figure 1B; note that *Trichoderma* has not yet sporulated over the surface of *Rhizoctonia solani* at the left side of the plate: photo from above at 12 days). The interaction with *Sclerotium rolfsii* was more complex, with some hyphae of this pathogen initially overgrowing wt *Trichoderma* after the two colonies met, but with a complete prevalence of wt over *S. rolfsii*, and a covering of the sclerotia formed in the oldest region of the *S. rolfsii* colony, later on. Δ*pacC* initially followed the same pattern as wt, eventually overgrowing *S. rolfsii*, yet was unable to cover the sclerotia. As seen in Figure 2, sporulation of wt (green conidia, arrows, left inset), but not sporulation of Δ*pacC* (right inset), was visible on the sclerotia. The ambient pH was similar in interactions with wt or with Δ*pacC* (note the similar distribution of indicator color: photos from below in Figure 2). At alkaline pH, *pacC*^c^ showed wild type virulence in confrontation with *Rhizoctonia solani*, despite its slightly reduced growth rate. At acidic pH, the growth rate of *pacC*^c^ was markedly reduced compared to wt (Figure 1A). Perhaps due to this slower growth, *pacC*^c^ was ineffective in confrontation assays against *S. rolfsii* (Figure 2). Both wt and *pacC*^c^ colony growth resulted in an alkalinization of the PDA medium, as has been observed previously for several *Trichoderma* species, when secondary nitrogen is available [36,47]. In comparison with the wt, the increase in pH was less extensive for Δ*pacC* (Figure 1B, 3 days, photo from below, bottom panel). Microscopic examination of the samples revealed mycoparasitic coiling in interactions of all three strains - wt, Δ*pacC,* and *pacC*^c^ - with *Rhizoctonia solani*. Furthermore, no morphological alterations or difference in the extent of coiling were evident when the three strains were compared (data not shown).

### Genome-wide study of PacC function

The phenotypes of the loss- and gain-of-function mutants in *pacC* that were studied here point to central roles for PacC in mycoparasitism. The PacC and pH-dependent transcriptomes provide insights into a variety of processes controlled by pH signaling, and into the mechanisms underlying pH homeostasis. To date, the transcriptomic consequences of pH signaling have only been studied in a relatively small number of fungal species [2,14,48,49]. The diversity of signal processing among fungal species suggests that a conserved regulatory factor can have different targets in different biological models. Thus, we reasoned that the *Trichoderma virens* transcriptome could provide insights into the role of pH signaling in fungal-fungal interactions. To this end, *T. virens* cultures were exposed to a one-hour alkaline or acidic pH step by transferring them to medium titrated to pH 8.4 or pH 4 (see Methods), and subsequently harvested.

**Figure 3:**
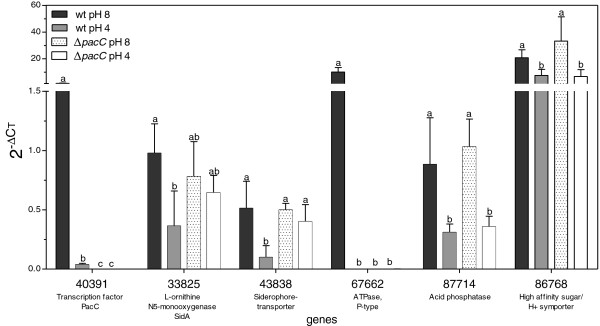
**Expression profiles of six candidate pH-regulated genes.** Expression of six of the genes listed in (Table 1) was followed by qPCR. Bars show the mean of 3 experiments; error bars indicate ± SD. Different letters (a, b, c) indicate significant differences between treatments according to ANOVA of the data for each gene (significance: post-ANOVA Tukey’s test, P<0.05).

Prior to performing microarray analysis, we chose 12 genes whose expression was previously reported to be regulated by pH in other fungi, and identified their orthologs in *T. virens* (Table 1). Expression of these genes in wt and Δ*pacC* was compared following exposure to the alkaline and acidic pH steps (we will refer to these as pH 8 and pH 4, respectively). The results we obtained for six of these genes are shown in Figure 3. As expected, *pacC* transcripts were not detected in the deletion mutant because the entire coding sequence was deleted (Additional file 3). In accordance with what has been reported for other fungi, we found that in the wt, exposure to alkaline pH resulted in an upregulation of *pacC* transcript levels. Of the genes listed in Table 1, the only one, other than *pacC,* to exhibit PacC-dependent expression was a P-ATPase gene, ID 67662. PacC-dependent expression was reported previously for the *Fusarium oxysporum* ortholog of this gene, *ENA1*[5]. We note that “protein ID” numbers are used throughout this report to designate both the gene and the predicted protein, because these identifiers conveniently lead to the complete information in the *T. virens* genome website (see Methods). Two genes were found to be upregulated by alkaline pH, but not via PacC: these were ID 87714 (an acid phosphatase) and ID 86768 (a high affinity glucose transporter). Acid phosphatase is a known acid-expressed gene in *Aspergillus nidulans*[3]. It is intriguing, therefore, that this gene was found here to be upregulated by the alkaline pH shift (or downregulated, perhaps, at the acidic pH where its activity would have been expected to be optimal). We found that two iron uptake-related genes: SidA (which catalyzes an early step in siderophore biosynthesis) and a predicted siderophore transporter were upregulated by the pH 8 step. Since Fe solubility decreases with increasing pH, it is possible that increased expression of siderophore biosynthesis-related genes may reflect an adaptation to limited iron availability. The other genes we examined (Table 1) did not show any significant dependence on pH. This is consistent with the existence of variations in pH and other signaling networks between different species (see also [43]). The results in Figure 3 show the similarity in the pH-regulation of *T. virens pacC* expression to that of its well-studied *Aspergillus nidulans* ortholog. In summary, these data demonstrated that the pH step signal was transduced to changes in gene expression, and we thus proceeded to a full-scale transcriptomic analysis.

**Table 1:** **Genes selected for initial studies of pH regulation in *T. virens***

**Protein ID**	**Sequence**	**Function**	**Species**
40391	s ACGCCGAGACGCTCTATGAAC	Transcription factor PacC	*T. virens*, this study
	as GGTGGTGCGACAAGAGTTCC		
33825	s CGAGACAGACCAGGGAGAGC	L-ornithine N5-monooxygenase SidA (2.5E-124)	*Aspergillus nidulans*
	as GAGTGTCGCTCAACCCATGA		
43838	s GTTGGGTACAACGGCTTGGA	Siderophore-transporter (9.02E-57)	*Aspergillus nidulans*
	as CAAGAAGGCGTTGGAGATGG		
111866	s CCGATTTGGTGGCTTCAGAT	Chitinase (0)	*Trichoderma harzianum*
	as ATTGGTGCCGACATCATTCC		
67662	as ATTGGTGCCGACATCATTCC	ATPase, P-type (0)	*Fusarium oxysporum*
	s GCTGACCTCCGACTGATTGAAG		
87714	as GCCAGTGGCATCATCAAAGA	Acid phosphatase (6.50E-95)	*Aspergillus nidulans*
	s GCCTTTGACCGCTTTGTTGT		
53036	as AGTTGGGCTCAGAAGGGTGA	Alpha-L-arabinofuranosidase (0)	*Aspergillus nidulans*
	s TTCGCCGAAGATGCTACGTT		
59192	as ATGTGTGAACACCGCCATTG	P-ATPase	*T. virens*
	s CTGGCCGTCATTCTCCTCAA		
	as GGGTTTCTGCCTTGACGGTA		
72838	s CCAGGCACCAAGAATAAGGTCAT	Xylanase (8.00E-76)	*Aspergillus nidulans*
	as GCCAGTGGATGGGTTGTAGG		
76958	s GTGTCATCTGGGTCGTTGGTT	Glucose permease (0)	*Trichoderma harzianum*
	as AGGGACCTGAGCAGATTCGAT		
81777	s GCACCAGCAAACCGGAAGC	Aspartyl protease (0)	*Trichoderma harzianum*
	as GTAACCGGTGGCAGTGAAGC		
86768	s GGTCTCGGTGTCGGTTTCG	High affinity sugar/H+ symporter (0)	*Aspergillus niger*
	as GCAAGAAGCGAGGAGGATACC		

Candidate genes whose expression might depend on pH and/or PacC were chosen from published studies in *Aspergillus nidulans*[28], *Fusarium oxysporum*[5] and *T. harzianum*[35]. A *T. virens* P-type ATPase (ID 59192) was already available from a differential library for transcripts expressed preferentially in cultures grown on autoclaved *R. solani* material. Protein ID numbers refer to the *T. virens* v1.0 sequence. E values (in parentheses) are from BLASTP searches of the *T. virens* genome with the corresponding genes indicated in the next column (species, GenBank accession).

#### Microarray analysis

**Figure 4:**
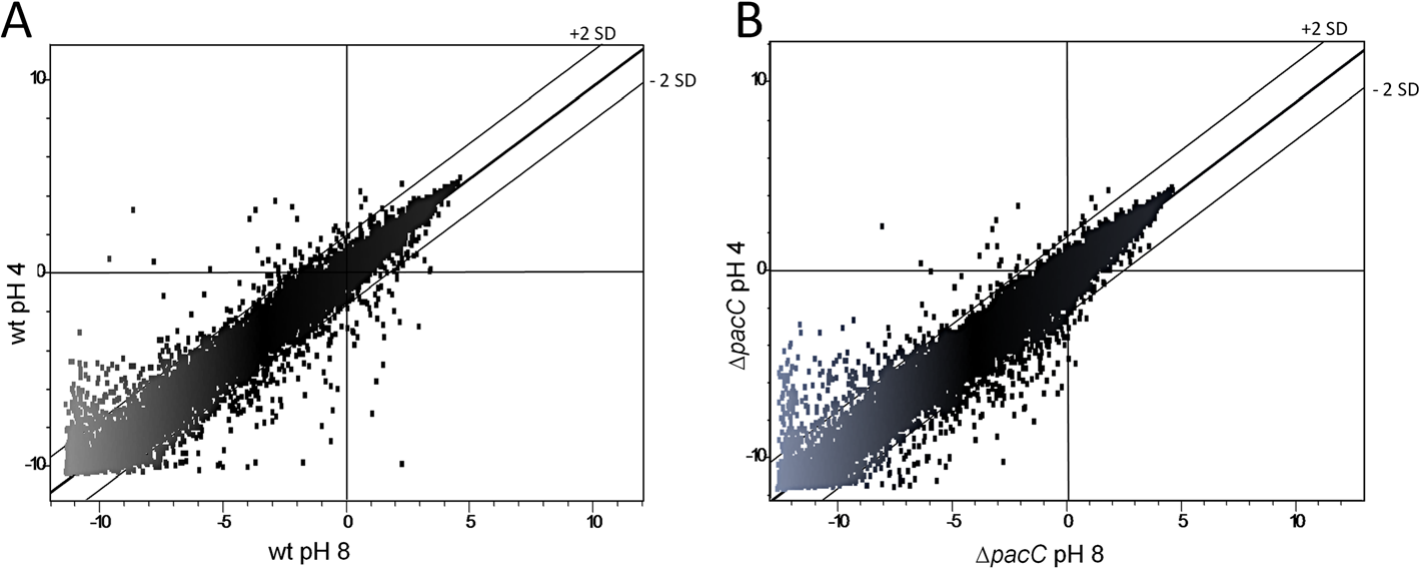
**Scatter plot representation of microarray data from wt (A) and *ΔpacC *(B) at pH 8 compared to pH 4.** Each dot represents the microarray signal of the particular gene. The best-fit linear trend and the ± 2 standard deviation (SD) lines (dashed) are indicated.

**Figure 5:**
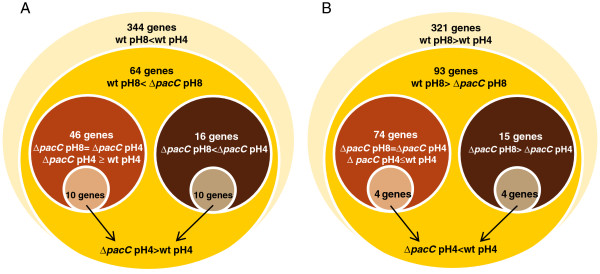
**Venn diagram of classes of significantly regulated genes.** The diagram shows subclasses of downregulated (**A**) and upregulated (**B**) genes for wild type and *ΔpacC*, and their overlap. > indicates greater transcript levels in the treatment shown at the left of the symbol; < indicates decreased transcript levels. The lists of genes in each class are given in Additional file 7.

Oligonucleotide microarrays were hybridized with probes synthesized from RNA samples from the pH 4 and pH 8 treatments. Plotting the signals from wt or *ΔpacC* at pH 8 against those at pH 4 (scatter plot in Figure 4) revealed that most of the signals were distributed within ± 2 standard deviation limits around the trendline. Treatment-dependent genes found below or above the trendlines deviate from what would be expected from random fluctuations in the transcript levels. Thus, this graphic analysis of the data shows that a large number of genes were regulated by the pH step treatments. A complete statistical analysis of the transcriptional profiles with LIMMA using a cutoff of >2-fold change and a p-value of *P* < 0.05 revealed that in the wt, more than 650 genes were differentially regulated in response to pH. The significantly regulated classes defined by LIMMA analyses are depicted in Figure 5. Of these pH-dependent genes, 157 were differentially expressed after exposure to the alkaline pH step in wt relative to *ΔpacC* (64 genes downregulated, Figure 5A, and 93 upregulated, Figure 5B). The 650 genes regulated by pH comprise ~about 5% of the 12,427 predicted protein-encoding transcripts, indicating that *T. virens* has a far-reaching transcriptomic response to external pH. PacC is responsible, either partially or entirely, for the pH regulation of 1% of all protein-encoding genes. This large number makes PacC a global transcriptional regulator in *T. virens*. NRG1 and SMP1, negative regulators which transduce the signal downstream of Rim101/PacC in yeast [48], were not found in the set of downregulated genes in our analysis.

#### Validation of microarray results by qPCR

**Figure 6:**
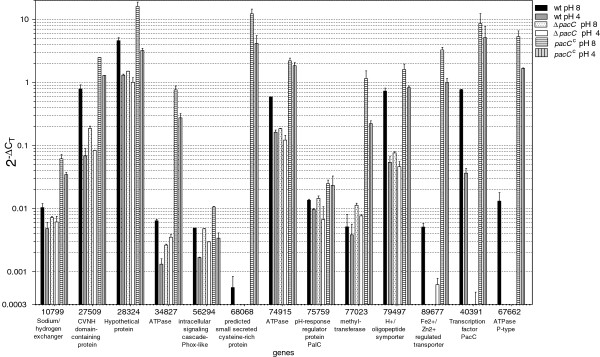
**Expression profiles of selected genes from the microarray data, measured by qPCR.** Expression profiles of 13 genes (Table 2) chosen from the microarray data were followed by qPCR. Error bars indicate ± SD for three experiments (biological repeats). The cutoff level of the y-axis was set by the highest signals obtained in no-template controls.

Two qPCR data sets were used for validation of the microarray data. The first set has been described above (Table 1, Figure 3). In Figure 3, data of qPCR amplifications from the same three independent RNA samples that we used to generate the probes for microarray analysis are presented. For the second qPCR data set, we performed an additional biological repeat under identical conditions, and the constitutively-active *pacC*^c^ mutant was also included (a total of 3 biological repeats). We then checked the expression of 13 candidate PacC-dependent genes (Table 2) using qPCR. The results are shown in Figure 6. In general, we found the regulation patterns determined by qPCR agreed well with the microarray hybridization data. As shown in Figure 6, the expression pattern of the panel of genes tested in the constitutively-active *pacC*^c^ strain (for both the pH values examined) was largely similar to that of the wt at pH 8. The expression level of most of these genes, however, was higher in *pacC*^c^ than in the wt. This suggests that activation of PacC in the wt does not reach saturation levels under ambient alkaline conditions, and that further activation is possible, and might occur in nature at unusually high pH values. Alternatively, since the constitutively active allele is present throughout growth whereas in wt the activation of PacC occurs only during the pH step, the response in the latter might then (depending on the lifetime of each transcript) be integrated over a relatively short time.

**Table 2:** Genes selected from microarray data for validation by qPCR

**Protein ID**	** Sequence**	**Gene homology**
10799	s CGACCGGCCAGGAGAGCAC	regulation of pH - Na/H exchanger
	as GGCTTGAACACGGGCTTGG	
27509	s CCCAGAACTACCGCCTTGAA	CVNH domain-containing protein
	as TCAACCCAGCTACCGTCCAC	
28147	s GCAAGAACATCCGCGACGAG	C-type lectin
	as CGCATAGCCTTCCTGAGCAT	
28324	s GCCAACGAAGAACGAAGCAG	Hypothetical
	as CTGATCGCCCTGTCCCATAA	
34827	s CCGAGTCTCTTGTTGCTGTCCTA	ATPase
	as TCGGAGCAGATGTTGGTGAC	
56294	s CCGCAGGTGAGAATCAGAGAGT	ATPase
	as CGTCTTCGTCTGTCGGTGTG	
68068	s TGAGGATCACTGGCATTGCTC	predicted small secreted cysteine-rich protein
	as GGTAGGTGGGAGATGGCACA	
74915	s TTCGTCATGGAGGCTGCTG	ATPase
	as ACCGCAGATGACACCGAAAT	
75759	s AATGATACGATGAACACACGACCT	pH-response regulator protein palC
	as GTGAATCTCACGGCCCGAAG	
77023	s TTCCACCTCGGCAAATATGATG	methyltransferase
	as TCCTGGCCGTATCAATGAAGA	
79497	s TCTCGTTGGTGCCTGGATTG	H+/oligopeptide symporter
	as CAGAGGCGACGAGGATAACG	
89677	s GCGCTGATACCTACTGCCTTGA	Fe2+/Zn2+ regulated transporter
	as ATCGGGATCTGCAGTGTCGT	
40391	s ACGCCGAGACGCTCTATGAAC	Transcription factor PacC
	as GGTGGTGCGACAAGAGTTCC	
67662	s GCTGACCTCCGACTGATTGAAG	ATPase, P-type
	as GCCAGTGGCATCATCAAAGA	
43392	s CCTCTGTAACCTCGATTCCAACG	peptidylprolyl isomerase A [PPIA] - normalization gene
	as AGCTCTGGCTCCTGGGTAGG	
77851	s CGTACTACAGCCGCTACCAGACC	ribosomal protein L5 [RPL5] - normalization gene
	as AGGCGGTACTTGGGAGCATT	
88010	s ACTTCAACGAGGCTTCTGGCAAC	Tubulin - normalization gene
	as CGGAACAGCTGGCCAAAGG	
91925	s AGGAAGAAGTTGCTGCCCTCGTCATCGACA	Actin - normalization gene
	as CCCATACCGATCATGATACCATGGTGACG	

Following microarray analyses, genes were chosen based on fold change and relevance. Protein ID numbers refer to the *T. virens* v1.0 database.

#### Classification of regulated transcripts

**Figure 7:**
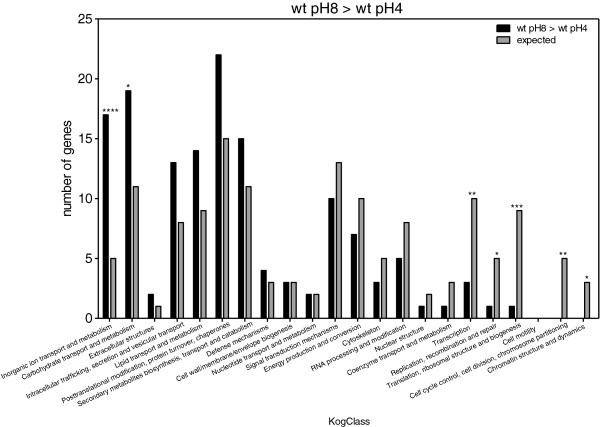
**KOG Classes represented in differently expressed gene lists.** wt pH8 > wt pH4. Asterisks indicate significant differences. X-axis (KogClasses) indicates the functional categories.

**Figure 8:**
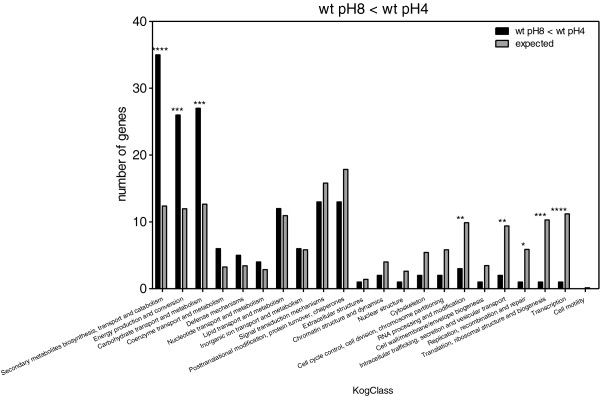
**KOG Classes represented in differently expressed gene lists.** wt pH8 < wt pH4. Asterisks indicate significant differences. X-axis (KogClasses) indicates the functional categories.

**Figure 9:**
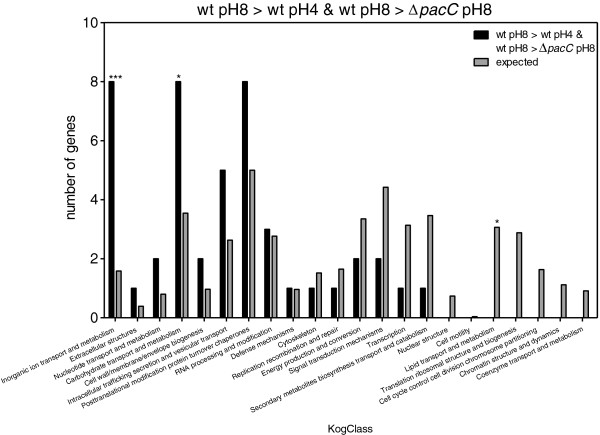
**KOG Classes represented in differently expressed gene lists.** wt pH8 > wt pH4 and wt pH8 > *ΔpacC* pH8. Asterisks indicate significant differences. X-axis (KogClasses) indicates the functional categories.

**Figure 10:**
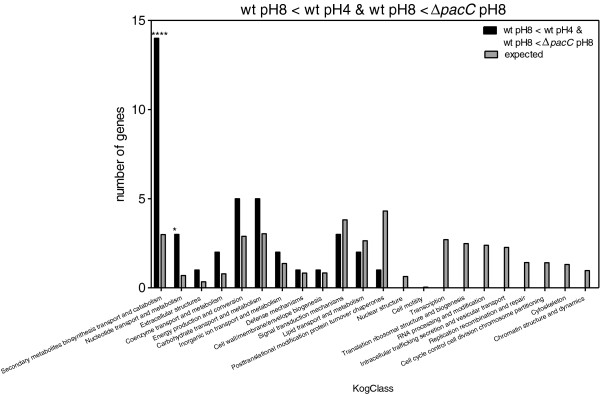
**KOG Classes represented in differently expressed gene lists.** wt pH8 < wt pH4 and wt pH8 <*ΔpacC* pH8. Asterisks indicate significant differences. X-axis (KogClasses) indicates the functional categories.

Enrichment in KOG classes was measured by comparing the expected number of genes representing each KOG class with the observed numbers. When we compared the genes that were upregulated in the wt at pH 8 as compared to pH 4 (Figure 7), we found that there was a clear enrichment in the expression of genes involved in carbohydrate and inorganic ion transport, metabolism, and “deficiency” in classes related to transcription, replication, translation, and cell cycle control. Comparison of genes that were downregulated in wt at pH 8 vs*. *wt at pH 4 (Figure 8) showed that there was an enrichment in genes involved in secondary metabolite biosynthesis, transport and catabolism; energy production and conversion; carbohydrate transport and metabolism. In addition, we found there were fewer genes than expected belonging to the following groups: transcription; translation, ribosomal structure and biogenesis; replication, recombination and repair; intracellular trafficking, secretion and vesicular transport; RNA processing and modification. PacC upregulated genes should meet two criteria: those whose expression is significantly greater in wt pH 8 vs. wt pH 4, and also significantly greater in wt pH 8 vs. *ΔpacC* pH 8. This set showed enrichment in carbohydrate and inorganic ion transport and metabolism (Figure 9). Conversely, PacC downregulated genes are those whose expression is significantly lower in wt pH 8 vs. wt pH 4, and in wt pH 8 vs. *ΔpacC* pH 8. This set showed enrichment in secondary metabolite biosynthesis, transport and catabolism, and in nucleotide transport and metabolism (Figure 10).

Secondary metabolite biosynthesis is finely tuned, and there is previous evidence for its regulation by ambient pH [45,50,51]. One of the proteins included in the PacC-dependent, down-regulated secondary metabolite-related gene class is a non-ribosomal peptide synthase, protein ID 37142. The gene coding for this protein, named *tex15*[52], is related to *ChNPS4* of *Cochliobolus heterostrophus*, which has a role in conidial surface hydrophobicity [53]. The *Alternaria brassicicola* ortholog of this gene is important for the development and integrity of conidia [54]. Inspection of the *T. virens* genomic region near *tex15* revealed a cytochrome P450 (protein ID 13148), which was also significantly down-regulated (Additional file 7). Additional members of this putative *ChNPS4*-like cluster were not significantly down-regulated (data not shown). Members of secondary metabolite biosynthetic clusters are known to be PacC-regulated in other fungi as well [50]. It would be rather surprising if all of secondary metabolism were to be downregulated at alkaline pH in *Trichoderma*. Two genes related to siderophore biosynthesis and import are indeed upregulated at alkaline pH in *T. virens* (Figure 3) and in other fungi as well (see Table 1). The SidA gene, ID 33825, is located near a predicted NRPS gene: ID 85582, Tex10 [52]. Tex10 is the ortholog of *C. heterostrophus NPS2*, and is responsible for the biosynthesis of the intracellular siderophore [55]. However, although Tex10 is clustered with *SidA*, it is not coregulated with it. The siderophore transporter ID 43838 shown in Figure 3 is clustered in the genome with ID 44273, a predicted NRPS gene showing 41% identity with *ChNPS6* of *C. heterostrophus*. The transcript corresponding to ID 44273 is co-regulated with the siderophore transporter ID 43838 (Figure 3). We note that there is no functional information for the gene corresponding to protein ID 44273. The most closely related NRPS to *ChNPS6* in *T. virens* is a different gene, which corresponds to protein ID 57567 [52], and unfortunately is not represented in the microarray probe set. *T. reesei* also has two paralogous *NPS6*-like genes [53].

Eight clearly-annotated genes encoding ion transporters were in the alkaline-upregulated, PacC dependent group (Additional file 8). These are of interest because of the importance of maintaining ion homeostasis, which is also tightly linked to pH homeostasis. The P-type ATPase Ena1 of *Fusarium oxysporum* (ortholog of *T. virens* ID 67662), for example, is known to be required for growth at alkaline pH and high salt [5]. ENA1 was one of the first targets of Rim101 (PacC) to be studied in *Saccharomyces cereviseae*[1]. Protein ID 89677 is annotated as a ZIP zinc transporter. These zinc transporters have been studied in fungal pathogens: for example, zrfA–C of *A. fumigatus* are regulated by pH and by available Zn levels [56]. Zrt1 of *Candida albicans* is important for virulence, possibly via the maintenance of cell wall integrity.

To test whether *ena1* is involved in the *pacC* related phenotypes, we proceeded to construct a knockout mutant, Δ*ena1*, in which this gene was replaced by the hygromycin B resistance cassette, using the split-marker procedure [46] as described above for *pacC*. Growth on acidic and alkaline pH and colony morphology of Δ*ena1* were normal. Likewise, Δ*ena1* performed similarly to wt in confrontation assays with *R. solani*, and with *S. rolfsii* (Additional file 9). It is not surprising that the deletion of a single gene did not account for the effect of a global regulator like PacC. Thus, we reason that either there may be redundant P-Type ATPase genes, or that a loss of a combination of factors may be required to account for the Δ*pacC* phenotype.

Some additional examples of pH and/or PacC-regulated genes are given in Table 3. An ortholog of DFG5, a cell-wall glycoprotein involved in tolerance to alkaline pH in *Candida albicans*, is upregulated 324-fold by alkaline pH in wt, but not in Δ*pacC*. In *S. cerevisiae*, expression of this gene depends on alkaline pH stress and on the cell wall integrity MAPK Slt2 [57], suggesting joint control of gene expression in response to high pH by PacC and by the cell wall integrity pathway. Protein ID 72478 is similar to GPR1/FUN34/yaaH, an ammonia exporter (AMET) gene which is upregulated by alkaline pH in *Colletotrichum gloeosporioides*[14]. To gain a more comprehensive biological interpretation it will be necessary to carry out a functional study of pH-regulated genes.

**Table 3:** Additional pH regulated genes with functional annotation

**Protein ID**	**Regulation in *T. virens***	**Homology**	**Species and regulation**
72478	upregulated at alkaline pH	GPR1/FUN34/yaaH ammonia exporter	upregulated at alkaline pH in *Colletotrichum*[14]
76718	upregulated at alkaline pH	glucose-repressible protein (GRG1)	upregulated at alkaline pH in *Colletotrichum*[14]
58111	upregulated at alkaline pH, PacC dependent	DFG5, a cell-wall glycoprotein involved in tolerance to alkaline pH	Upregulated at alkaline pH in *Candida albicans*[58,59]; in *S. cerevisiae*, expression depends on alkaline pH stress and the cell wall integrity MAPK Slt2 [57]
83099	downregulated at alkaline pH	like NAD^+^ epimerase/dehydratase family, EPME	alkaline pH downregulated in *Colletotrichum*[14]
73483	downregulated at alkaline pH (2.5 downregulation trend, though below the level of significance for our cut-off criteria)	high affinity glucose transporter	alkaline pH downregulated in *Colletotrichum*[14]

Secondary metabolite biosynthesis genes whose expression is not necessary at high pH could be expected to be downregulated. This would occur if interaction with particular soil microorganisms did not pose a threat (or if the soil microorganisms targeted by a particular metabolite were not available as hosts for the mycoparasite) in an alkaline environment. In contrast, siderophores are necessary for Fe uptake, which becomes increasingly difficult at higher pH. The export of ammonia increases pH. The increase in the level of the transcript for an ammonia transporter (Table 3) suggests that ammonia export is upregulated with increasing pH. This would indicate a quorum-sensing like feedback mechanism, in which local alkalinization is self-reinforcing. In this respect, it would be interesting to compare a foliar pathogen (*C. gloeosporioides*) with a rhizosphere resident (*T. virens*).

#### *Predicted* PacC *binding sites*

**Figure 11:**
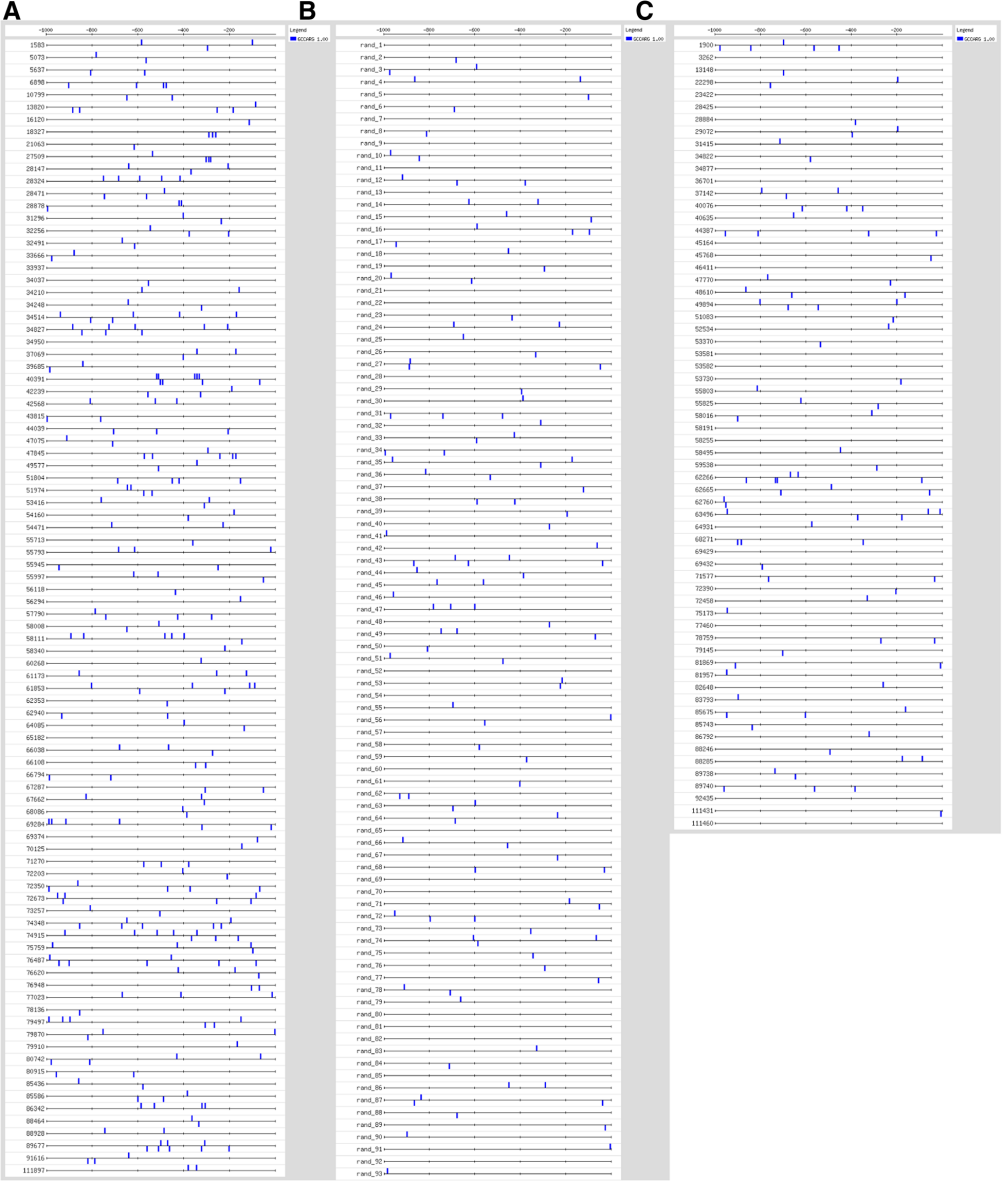
**Identification of predicted binding sites for PacC.** Binding sites for PacC in the 1 kb upstream of the start codon were identified using Regulatory Sequence Analysis Tools (RSAT, http://rsat.ulb.ac.be/). **A**. Promoters of genes upregulated in wt pH 8 *vs.* wt pH 4 and underexpressed in *ΔpacC* vs wt pH 8. **B**. Random promoter sequences from *T. virens.***C**. Promoters of genes downregulated in wt pH 8 *vs.* wt pH 4 and underexpressed in wt *vs. ΔpacC* at pH 8.

In *Aspergillus nidulans*, PacC binds 5-GCCARG-3 sequences upstream of pH-responsive genes, and can activate or repress transcription of those genes [41]. To determine whether this target sequence could be conserved in *T. virens*, we quantified the number of predicted PacC binding sites in the 1 kb regions upstream of the start codon of each of the PacC-regulated genes (Additional files 8, 10). We found that there were 3.1 instances per 1 kb for genes upregulated via PacC (shown schematically in Figure 11A). The frequency at which this site appeared in random promoters, 1.3 instances per 1 kb (Figure 11B), is clearly much lower. No such enrichment was found for the PacC-downregulated genes (1.5 instances per 1 kb, Figure 11C). This suggests a different mode of regulation, i.e., direct for activation, and indirect for repression, of the PacC target genes.

**Figure 12:**
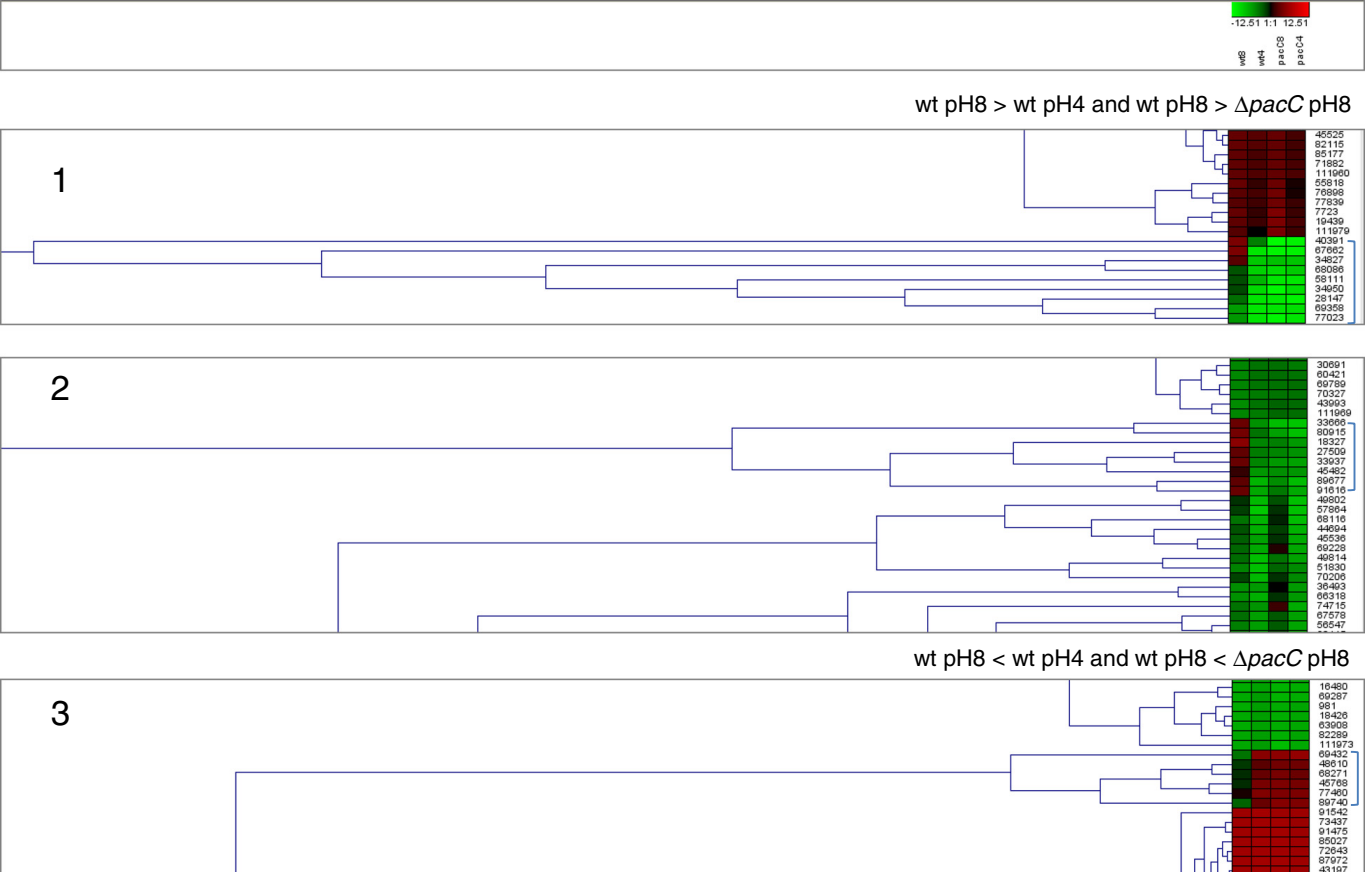
**Representative results of cluster analysis.** Average microarray signals were clustered (Genesis, http://genome.tugraz.at/).

Cluster analysis performed on the averaged microarray data for each experimental treatment indicates a well-defined group that is upregulated at alkaline pH in wt but not in Δ*pacC* (Figure 12, Cluster 1); this cluster includes *ena1* (see above). *pacC* itself also belongs to Cluster 1: *pacC* transcript abundance is increased at pH 8 in wt. There is a small group of genes whose expression is PacC-dependent at pH 4 (Table 4). Acidic pH is not expected to promote proteolytic activation of the unprocessed, long form of PacC. This suggests the existence of a novel mechanism which could be studied by promoter analysis of this group. An additional group of genes responds to pH essentially as the wild type does, but their expression level is lower (Table 4). Thus, for this gene group, a PacC-dependent process regulates overall expression level. This regulation could consist of a gating process in which PacC increases the expression of genes that are initially regulated by a different transcription factor.

**Table 4:** Groups of genes with expression patterns that have not been studied previously

**Acid regulated**				**Gating process**			
***ΔpacC *pH4>wt pH4**			***ΔpacC *pH4<wt pH4**	***ΔpacC *pH8<*ΔpacC *pH4**		***ΔpacC *pH8>*ΔpacC *pH4**	
69432	72458	53730	33937	91616	74915	62760	88246
52534	85743	37142	33666	18327	62940	44387	71577
48610	36701	49894	18327	72350	79910	58495	13148
34822	88246	82648	74915	51804	55793	34822	79145
53582	13148		85436	49577	85436	53582	53581
89740	79145		79497	16120	79497	72390	92435
85675	53581		66108	88928	66794	81957	53730
78759	92435			66038		85743	37142

Acid-regulated, PacC dependent (left); expression gated by PacC at both pH 4 and pH 8 (right).

*Trichoderma* spp. are widely used as biofungicides. Even though approximately 60% of all registered biofungicides are *Trichoderma*-based, the market size of biopesticides is only a small fraction of the total pesticide market, which is largely dominated by chemicals [31,60]. Bioefficacy of *Trichoderma*, like any other biocontrol agent, is bound to be strongly influenced by environmental factors. One of the major limitations of applying *Trichoderma* in agricultural settings is its inability to operate in alkaline conditions, since the pH optima for *Trichoderma* growth, development, and activities is in the range of 4–6 [61]. In this respect, an understanding of the pH signaling pathways might be instrumental in improving the bioefficacy of *Trichoderma* spp. Thus, this study was carried out with the goal of elucidating the role of the pH-responsive transcription factor PacC and the gene regulation network mediated by this transcription factor in the commercially used biofungicide *T. virens*. The phenotypes of loss- and gain-of-function *pacC* mutants studied here reveal the importance of this signaling pathway in development, and in the mycoparasitic interaction of *T. virens* with two important fungal pathogens, *R. solani* and *S. rolfsii*. The present study also indicates that pH regulation does not depend entirely on PacC, since we found a number of up- and downregulated genes whose dependence on pH was similar in the Δ*pacC* mutant and wild type (despite the fact that steady-state growth of the mutant is very slow at pH 8). This result points to the possible existence of a pH-sensing mechanism other than the PacC pathway, by which normal regulation takes place during the one-hour alkaline pH step, despite its adverse effect on the growth rate. It will be interesting to identify such alternative pathways. The pathway for transduction of pH signals in *A. nidulans* has been described as “mechanistically dissimilar to all other known eukaryotic signal transduction pathways” [62]. This makes it an attractive target for antifungals, as has been proposed, for similar reasons, for the two-component signaling pathways of bacteria [63]. In biocontrol, however, growth of the (mycoparasite) pathogen is beneficial, so the reasoning usually applied to host-pathogen interactions would need to be reversed. Strains could be selected for growth at different ambient soil pH or for growth in the rhizosphere of different crop plants. Detection of ambient pH through PacC and other mechanisms, and the resulting ability to maintain pH homeostasis, are likely general requirements for a mycoparasite to attack the host, explaining the loss of biocontrol ability in *T. harzianum pacC* mutants [35]. Genetic manipulation of the global pH-dependent regulator PacC or some of its downstream target genes may be relevant for optimization of biocontrol strains according to the local environment, crop plant, and fungal host.

## Conclusions

Our genome-wide analysis of PacC and pH regulation identified some genes that are well-known targets of regulation by pH, for example *pacC* itself and *ena1*. We also found a large number of novel genes that are promising candidates for construction of loss-of-function mutants. Several hypotheses can be proposed to explain why pH (both PacC dependent and independent) regulation is important for mycoparasitism: pH homeostasis in the face of host-mediated changes in ambient pH, a signal provided by the host, and programming of expression of hydrolytic enzymes to be expressed near the pH optima for their activity. The results, while compatible with all these mechanisms, also indicate a more complex picture. For example, several genes encoding enzymes predicted to have acid pH optima are expressed at alkaline pH. Thus, an initial alkaline pH signal may anticipate varying pH during the fungal-fungal interaction. Measurement of local pH at the microscopic level, in real time during the interaction, could provide further insight. We anticipate that gene knockout and overexpression of candidate genes identified here will be helpful in improving the pH adaptability of the most popular biofungicides, i.e., *Trichoderma* spp.

## Methods

### Fungal strains and culture conditions

*T. virens*[38,64] is deposited as IMI 304061. Local isolates of *R. solani* and *S. rolfsii* were kindly provided by Dr. Ada Viterbo, Faculty of Agriculture, Hebrew University of Jerusalem, Rehovot. Wild type *T. virens* and *R. solani* were grown on potato dextrose agar (PDA; Difco) at room temperature unless otherwise indicated. Complete medium (PDYC) contained 24 g/l potato dextrose broth, 2 g/l yeast extract and 1.2 g casein hydrolysate (all from Difco). For long-term storage, a dense conidial suspension in PDYC, 20% glycerol was stored at −70°C. For hygromycin B selection, PDA was prepared with 100 μg/ml hygromycin B (Calbiochem). For phenotype assays on different pH-buffered media, the following buffers were used: for pH 4 and 5 - potassium hydrogen phthalate (Sigma-Aldrich), for pH 6 - KH_2_PO_4_, for pH 7, 8 and 9 -Trizma base (Sigma-Aldrich). For visualization of pH changes, Phenol Red (Riedel*-*de Haën) was added to the plates (final concentration 0.01%). The growth rate of the mutants relative to the wild type was determined by placing a 5-mm-diameter mycelial disk of the fungus in the centre of a PDA plate and measuring the colony diameter at the indicated times. For confrontation assays, *T. virens* was co-inoculated with host fungi by placing 5-mm-diameter mycelial discs at the opposite sides of PDA Petri dishes. Overgrowth of the host by the different *T. virens* strains is evident from the production of green conidia and the disappearance of the normal morphology of the host colony. For pH step treatments, shake cultures in PDB (initial pH 4.5) were grown for 3 days, and reached pH 5.2-5.9. The mycelia were collected by filtration and transferred to fresh medium titrated to either pH 8.4 or pH 4. Measured immediately (within minutes) after the addition of mycelium, the pH was 7.7-7.9. At the end of harvesting, the pH was 6.7-6.9. In the acidic direction, there was only a slight upward shift (less than 0.1 unit) upon addition of mycelium, and at most by 0.4 units after the experiment.

### RNA isolation

Total RNA was isolated from mycelia ground in liquid nitrogen with Tri-reagent (Molecular Research Center, Cincinnati, OH) according to the manufacturer’s protocol. Alternatively, mycelia were lysed by homogenization together with Tri-reagent in a Mini-bead Beater (Biospec Products, Bartlesville, Oklahoma), using 0.2 g of 0.5 mm-diameter zirconia/silica beads (BioSpec Products). RNA yield was evaluated using a NanoDrop ND-1000 spectrophotometer (NanoDrop, Wilmington, DE). Samples for microarray analysis were further evaluated using the Agilent 2100 Bioanalyzer and RNA 6000 Nano kit (Agilent, Santa Clara, CA) according to the manufacturer’s instructions. Samples with electropherograms exhibiting sharp 18S and 28S rRNA peaks and showing no evidence of degradation were retained.

### Real time qPCR

For cDNA synthesis, 2 μg of RNA were used for reverse transcription with random primers following the protocol supplied with the High Capacity cDNA Reverse Transcription Kit (Applied Biosystems). Abundance of transcripts was measured by real-time qRT-PCR reactions performed in an Applied Biosystems 7000 cycler; approximately 15 ng of cDNA were used as template. The 15 μl reaction volume included 7.5 μl of 2XABsolute SYBR Green ROX MIX (ABgene, Surrey, U.K.) and 75 nM final concentration of specific primers for the gene of interest. Assays were run in duplicate or triplicate, using the following protocol: initial activation at 95°C for 10 min; 40 cycles of 95°C for 15 s, 60°C for 60 s, followed by a gradual increase in temperature from 60°C to 95°C during the dissociation stage. Tables 1 and 2 detail the genes validated by qPCR and assay conditions. The transcript abundance was calculated using the ABI software DataAssist. Prior to quantitative analysis, a standard curve was constructed using serial dilutions of RT product and the efficiency of each primer set was determined using the equation [(10^(−1/slope)^-1)·100]. Efficiencies near 100% were required to include the qPCR assay in array validation. For balancing the amount of cDNA, the following were tested for stable expression across all experimental treatments using DataAssist software (ABI): tubulin (protein ID 88010), peptidylprolyl isomerase A (PPIA, protein ID 43392), actin (protein ID 91925) and ribosomal protein L5 (RPL5, protein ID 77851). Tubulin, PPIA and L5 were chosen as “housekeeping” genes.

### Gene replacement: Δ*pacC*

A fragment of the *pacC* gene was cloned by amplification with degenerate primers, as described in the Results. We used a PCR-based genome walking method (Universal Genome Walker kit, Clontech) to clone the unknown (prior to assembly of the *T. virens* sequence at the Joint Genome Institute) genomic DNA sequences adjacent to our known sequence. Genomic libraries were prepared and amplifications were done according to the manufacturer’s instructions. The deletion construct was made by replacement of the coding region with the selection marker gene for hygromycin resistance, hygromycin phosphotransferase (*hph*) under the control of the *Aspergillus nidulans* TrpC promoter and TrpC transcription termination signals (Additional file 11). 1.5 kb upstream and 1 kb downstream flanking sequences of the *pacC* gene were amplified with the following primer pairs: (5′ flank s and 5′ flank as primers) and (3′ flank s and 3′ flank as primers) respectively (Additional file 12), by PCR using BIO-X-ACT high fidelity enzyme (Bioline). The resulting 1.5 kb upstream PCR fragment was cloned into pUC57 (Fermentas), and the resulting plasmid digested with *Acc*65I and *Eco*RI. The 1 kb downstream PCR fragment was digested with *Not*I and *Eco*RI. The Hph cassette was excised from pUC-ATPH [65] with *Acc*65I and *Not*I. For *pacC* replacement, pUC57 plasmid containing the upstream fragment was ligated in one step using T4 DNA ligase (NEB) to the downstream flank and Hph cassette fragment (Additional file 11). Transformation to HIT DH5α (UBI) competent cells was performed according to the manufacturer’s protocol. Plasmid DNA was isolated from bacterial cultures using the Qiagen Plasmid Mini Kit. Linear DNA for transformation was prepared by cleavage of the plasmid with *Eco*RI (New England Biolabs).

### Split-marker method: *pacC*^*c*^

The *pacC*^*c*^ mutant was constructed using the split-marker method [46,66]. A linear DNA construct was made by overlapping PCR reactions. The construct, upon integration into the genome, inserts the hygromycin selectable marker flanked from one side by the truncated *pacC* gene (constitutively active) ended with an inserted TAA stop codon, and 3′ UTR [46] (Additional file 4). Reactions with primer pairs 1 and 2 (Additional file 13) were carried out using *T. virens* genomic DNA as template. Reactions with pairs 3 and 4 were carried out using pBlueScript-Hyg (derived from pUCATPH [65]) plasmid DNA as template. The product obtained with primer pair 3 or pair 4 is part of the gene (*hph*) encoding hygromycin phosphotransferase. The second round of PCR joins the 5′ flank (truncated *pacC* gene) or 3′ flank with the partial *hph* coding sequences amplified in the first round, by annealing of the complementary sequence present in the reverse primer of pair 1 or the forward primer of pair 2, respectively. To construct the 5′ side of the final sequence, the products of pairs 1, 3 were used as template for amplification with primers FP1 and NLC37; for the second half, the products of pairs 2 and 4 were used as template for amplification with primers NLC38 and RP2. The two final products were integrated into the *T. virens* genome by double-crossover recombination, resulting in reconstruction of the complete hygromycin resistance cassette and replacement of the 3′ coding region of *pacC*. Fungal protoplasts were prepared as described below; transformants were selected for hygromycin resistance, and tested for homologous integration by amplification with primer pairs 5 and 6 (Additional file 13) and others (Additional file 3).

### Transformation

Transformation was performed essentially as described by [67], optimized for *T. virens*. The protoplasts were released from germinating conidia by 2 to 2.5 h of digestion at 30°C with gentle shaking (70 rpm, rotary shaker) in an enzyme mixture which consisted of 0.2 g of β-D-glucanase (InterSpex, San Mateo, CA - we note that InterSpex products are no longer available; replacements are currently being tested and calibrated), 0.4 g of Driselase (InterSpex), and approximately 2 mg of chitinase (Sigma, catalog number C6137). The mixture was stirred for 5 min at room temperature in 70 ml of 0.7 M NaCl, centrifuged for 10 min at 8,000 rpm (Sorvall SS34 rotor), and sterile filtered (0.22-μm-pore-size filter). Protoplasts were counted, and mycelial fragments were removed by filtering through three layers of sterile gauze followed by one layer of Nytex 50 monofilament nylon mesh. Protoplasts were washed in STC (sorbitol, 1.2 M; Tris pH 7.5, 10 mM; CaCl2, 50 mM). About 10^8^ protoplasts were taken for transformation with 20 μg of plasmid DNA or linearized fragments for double-crossover integration. Selection was on 200 μg/ml of hygromycin B in PDA. Single spore isolates were obtained from transformants that formed conidia.

### Acid/alkaline pH conditions

For each line (wt, *ΔpacC* and *pacC*^*c*^), spores were collected from a 4-day old culture growing on a 90 mm PDA Petri dish (for mutants, PDA amended with 100 μg/ml Hygromycin B) and 10^7^ spores were inoculated into 100 ml potato dextrose broth (PDB; Difco) in six 250 ml Erlenmeyer flasks (three repeats for each pH). After 3 days of shaking at 170 rpm at 29°C under continuous light, the medium in each Erlenmeyer flask was replaced with fresh PDB at pH 4 or pH 8 (the pH was adjusted with HCl and NaOH, one hour before the experiment started), by filtering the mycelium onto one layer of Nytex 50 monfilament nylon mesh. The mycelium was immediately transferred into PDB at the desired pH, and the culture returned to shaking. After 1 hour, each mycelial sample was vacuum-harvested on a Buchner funnel and frozen in liquid nitrogen.

### Microarrays and sample preparation

A custom microarray was designed (Genotypic, Bangalore, India) from the complete set of filtered transcript models (*Trichoderma virens* v1.0, JGI http://genome.jgi-psf.org/Trive1/Trive1.home.html, [39]) and printed as 15 K arrays (Agilent, Santa Clara, CA, USA). To 1 μg total RNA, we added RNA from a spike-in kit (Agilent). cDNA synthesis was primed with oligo dT, and the double-stranded template was used for amplification and labelling by *in vitro* transcription using the MessageAmpII kit from Ambion (Austin, TX, USA). Amplified RNA (aRNA) was labeled with Cy3 and hybridized onto the custom microarrays. Microarray hybridization and washing steps were performed following the Agilent protocol for single-channel arrays. The arrays were scanned at 10% laser power to avoid signal saturation. Agilent’s Feature Extraction software was used to extract the data.

### Normalization of the expression data

Microarray signals were normalized to allow comparison of samples with different RNA amounts, using the spike-in data. First, the log_10_ microarray data were normalized so that signals for one of the spike-ins (E1A_r60_a20) with a log relative concentration of 3.83 had the same values across all samples. Next, as described previously [68,69], the log_10_ expression data were normalized by linearly interpolating to concentrations using the ten spike-in measurements for each sample and subsequently normalizing to the 75th percentile signal intensity. To summarize the replicate data for each gene, the mean for the three values (three biological replicates for each treatment) was computed. The complete data set and array platforms are provided in Additional file 14.

### Statistical analyses

Statistical analyses for qPCR were performed with the GraphPad Prism software version 5.00 (GraphPad Software, San Diego, California, USA, demo version). Unless otherwise indicated, the threshold level chosen for comparison of means was P<0.05 by Student’s t-test (one-tailed, non-paired, equal variance). Adjusted P values for microarray data were calculated using LIMMA implemented in R, and the cut-off was at an adjusted P value <0.05. Cluster analysis was done using Genesis, http://genome.tugraz.at/.

## Competing interests

The authors declare that they have no competing interests.

## Authors’ contributions

NT carried out molecular genetic studies, did the microarray experiments, participated in the design of the study and helped to draft the manuscript. ML participated in planning the microarray experiments, microarray hybridization, and carried out data analysis. PKM established the genetic framework to study *T. virens* and identified PacC as a target for study. BAH conceived the study and helped draft the manuscript. All authors read and approved the final manuscript.

## Supplementary Material

Additional file 1 **List of primers not included in Tables** 1**and **2**.** The primers listed here are referred to in the text. Degenerate primers, gene-specific primers (GSP) and adaptors were used to clone and sequence *pacC* prior to release of the genome sequence. Actin primers were used for normalization (housekeeping gene); “standard” primers were used in construction and validation of transformants. In the degenerate primers, I indicates inosine. Click here for file

Additional file 2 **Alignment of PacC protein from *T. virens* IMI 304061 and *T. virens *Gv29.8, the published reference strain.** Protein sequence alignment. Click here for file

Additional file 3 **Verification of double-crossover events in transformants.** This is a set of data confirming gene successful double crossover integration events. The linear map shows the *pacC* genomic region, with the relevant genes and markers indicated. Primer names are shown, with their locations and directions indicated by small arrows. PCR products are shown in the gel images; the source of the template DNA is shown in bold text below each lane, and the primer pairs used for the amplification are noted below the image, referring to the corresponding set of lanes. The primer names are also listed in the table (Expected sizes of PCR products from mutant validation), along with the predicted ampicon sizes. Click here for file

Additional file 4 **Split-marker gene replacement strategy.** This figure illustrates the split-marker strategy. In the first round of PCR reactions, the flanking regions are amplified; in the second round, they are each fused to part of the hygromycin resistance cassette (HYG). For primer sequences see Additional file 14; the strategy and diagram are adapted from [46]. Click here for file

Additional file 5 **Growth rate of wild type and *ΔpacC.*** This graph shows growth rate of the *ΔpacC* strain relative to the wild type. Growth was measured by placing a 5-mm-diameter mycelial disk of the fungus in the center of a PDA plate and measuring the colony diameter at the indicated times. Values represent an average of 4 replicates. Error bars represent the standard deviation. Click here for file

Additional file 6 **Morphology of wild type and *ΔpacC *mutant *T. virens*.** This figure is a photo of cultures, showing colony morphology. A 5-mm-diameter mycelial disk of the fungus was inoculated in the center of a PDA plate. The plates were incubated in the light and dark and colonies were photographed at the indicated times. Click here for file

Additional file 7 **Lists of differentially expressed genes from statistical analysis (Figure** 5**).** List of protein ID numbers for each category of the genes shown in Figure 5 of the main text. Click here for file

Additional file 8 **Expression profiles of PacC-dependent, alkaline pH upregulated genes, and available annotation.** Details of regulated genes are given here. Secreted proteins are indicated by a + if predicted at a SignalP score of at least 0.7. The number of predicted PacC binding sites (GCCARG, see text) in the 1 kb region upstream of the start codon is given in the last column. Expression profiles (fold expression at pH 8 compared to pH 4, and wt compared to Δ*pacC* at pH 8) are given. Annotation by homology: entries are best hits by BLAST, E values are given in the following column. Click here for file

Additional file 9 **Mutants in P type ATPase *Δena1 *are similar to wt in plate assays for growth and confrontation.** Photos of cultures grown on PDA plates, taken at 11 days from above to show overgrowth and sporulation (A), or below to show pH indicator color (B). The wt and *Δena1* strains were grown in confrontation with two plant pathogens. Click here for file

Additional file 10 **Expression profiles of PacC-dependent, acid pH upregulated genes, and available annotation.** Details of regulated genes are given here. Secreted proteins are indicated by a + if predicted at a SignalP score of at least 0.7. The number of predicted PacC binding sites (GCCARG, see text) in the 1 kb region upstream of the start codon is given in the last column. Expression profiles (fold expression at pH 8 compared to pH 4, and wt compared to Δ*pacC* at pH 8) are given. Annotation by homology: entries are best hits by BLAST, E values are given in the following column. Click here for file

Additional file 11 **Disruption of *pacC *by homologous integration.** The diagram shows the scheme used for replacement of the entire *pacC* coding sequence. HYG indicates selectable marker cassette (for full details, see Methods, main text). Click here for file

Additional file 12 **Primers for gene knock-out.** Sequences and names of the primers used for knock-out of *pacC* and confirmation of the integration event are given. Click here for file

Additional file 13 **Primers for split-marker gene replacement strategy.** This is a primer list for split-marker gene replacement. In the Table, lower case indicates sequences that are not complementary to the template in the first PCR step. In the second PCR step, these sequences, which are complementary to the ends of the selectable marker, allow joining of the fragments. Click here for file

Additional file 14 **Complete microarray data.** The complete microarray data are provided as a comma delimited file (Excel compatible). The first column gives the Protein ID. This number, when entered into the “search” box in the *T. virens* v1.0 web page, leads conveniently to all the available gene model, transcript and protein information. The second column gives the number of (identical) oligonucleotides spotted on the microarray for this gene. The following columns give the signal values for each independent experiment, after background subtraction and normalization (see Methods, main text). Click here for file
