# Supplementary material for: PacC and pH–dependent transcriptome of the mycotrophic fungus Trichoderma virens
Source: BMC Genomics. 2013 Feb 28;14:138. doi: 10.1186/1471-2164-14-138 (PMC3618310; doi:10.1186/1471-2164-14-138)
Supplement: Additional file 1 — List of primers not included in Tables 1and 2. The primers listed here are referred to in the text. Degenerate primers, gene-specific primers (GSP) and adaptors were used to clone and sequence pacC prior to release of the genome sequence. Actin primers were used for normalization (housekeeping gene); “standard” primers were used in construction and validation of transformants. In the degenerate primers, I indicates inosine. [file 1471-2164-14-138-S1.pdf]

Additional file 1 - List of primers not included in Tables 1 and 2

The primers listed here are referred to in the text. Degenerate primers, gene-specific primers (GSP) and adaptors were used to clone and sequence *pacC* prior to release of the genome sequence. Actin primers were used for normalization (housekeeping gene); “standard” primers were used in construction and validation of transformants. In the degenerate primers, I indicates inosine.

Comments	Primer	Sequence
pacC degenerate primer	PacFout	GA(C/T) GA(C/T) III GTN (T/C)TI GTN CGN CC
	PacFnest	AA(A/G) CGN CGN CA(A/G) (T/A)TI GA(C/T) CC
	PacRout	T NCC NCC III (A/G)TA NCG NCG NCG
	PacRnest	TT (T/C)TC (A/G)TA IAT NGT (A/G)TC (C/T)TG CAT (C/T)TG
GSP1 3’ flank	3pacC_out	TCC CTG CTC CCT CTT CAC AAT GCT CTT
GSP2 3’ flank	3pacC_nest	CAG TAC TAT TTG CCT ATG CCG AAT GCG A
GSP1 5’ flank	5pacC_nest	CGATCTGAGCATAGGAAGTCGGATC
GSP2 5’ flank	5pacC_out	TCGGTTGGGGAAAGTAAGATTCAGT
Adaptor Primer 1	AP1	GTAATACGACTCACTATAGGGC
Nested Adaptor Primer 2	AP2	ACTATAGGGCACGCGTGGT
T. virens actin s	TVact_s	GTCAGGTCATCACCATCGGCAACG
T. virens actin as	TVact_as	GCTGGAAGGTGGACAGGGAGGC
Standards	M13reverse	GGAAACAGCTATGACCATG
	M13-(20)forward	TGTAAAACGACGGCCAGT
	PtrpC	GGTCGTTCACTTACCTTGCTTG
	TtrpC	GGTGTTCAGGATCTCGATAAG
