# Supplementary material for: PacC and pH–dependent transcriptome of the mycotrophic fungus Trichoderma virens
Source: BMC Genomics. 2013 Feb 28;14:138. doi: 10.1186/1471-2164-14-138 (PMC3618310; doi:10.1186/1471-2164-14-138)
Supplement: Additional file 2 — Alignment of PacC protein from T. virens IMI 304061 and T. virens Gv29.8, the published reference strain. Protein sequence alignment. [file 1471-2164-14-138-S2.pdf]

Additional file 2 – Alignment of PacC protein from *T. virens* IMI 304061 and *T. virens* Gv29.8, the published reference strain.

The alignment was generated using ClustalW (http://www.ebi.ac.uk/Tools/msa/clustalw2/), default ClustalW colors are shown, indicating amino acid physicochemical properties.

```
IMI             MSAQVPDQAALSPSHDTKSASPTDASSTTSPNGSTSNNGSSASSNAQVSPSLGDAIDAPR 60
Gv29.8          MSAQVPDQAALSPSHDTKSASPTDASSTTSPNGSTSNNGSSASSNAQVSPSLGDAIDAPR 60
                ************************************************************

IMI             QLVCRWNQCGQKFTNAETLYEHICERHVGRKSTNNLSLTCQWNSCRTTTVKRDHITSHIR 120
Gv29.8          QLVCRWNQCGQKFTNAETLYEHICERHVGRKSTNNLSLTCQWNSCRTTTVKRDHITSHIR 120
                ************************************************************

IMI             VHVPLKPHKCEFCGKSFKRPQDLKKHVKTHADDSVLSRPGQDSQPGMNYRPQAPKPPSYY 180
Gv29.8          VHVPLKPHKCEFCGKSFKRPQDLKKHVKTHADDSVLSRPGQDSQPGMNYRPQAPKPPSYY 180
                ************************************************************

IMI             DHNGQMRSPVTGFPQPHAGGYYAPQPSTNYGLYFNQQHVNPAPRTEHIGYSASYDRKRTQ 240
Gv29.8          DHNGQMRSPVTGFPQPHAGGYYAPQPSTNYGLYFNQQHVNPAPRTEHIGYSASYDRKRTQ 240
                ************************************************************

IMI             AHEMVDDFFGSAKRRQIDPTSYAQIGRSLLPLHNALAMPSGPMAATESYFPQPTGHAVGH 300
Gv29.8          AHEMVDDFFGSAKRRQIDPTSYAQIGRSLLPLHNALAMPSGPMAATESYFPQPAGHAVGH 300
                *****************************************************:******

IMI             AVVHAAPAPTPTQNPLAQQYYLPMPNARTQKDLIQLDQMLGQMQDTVYETAPQITAGMHP 360
Gv29.8          GVVHAAPAPAPTQNPLAQQYYLPMPNARTQKDLIQLDQMLGQMQDTVYETAPQITAGMHP 360
                .********:**************************************************

IMI             HDSQFAGYRSTPSPTTLHRGPGAMHVAPDGYHQPVSAANMASPLTAISSTGTPAVTPPSS 420
Gv29.8          HDSQFAGYRSTPSPTTLHRGPGAIHVAPDGYHQPVSAASMASPLTAISSTGTPAVTPPSS 420
                ***********************:**************.*********************

IMI             ALSYTSGHSPSPSASSGFSPQSRHSSTASSIMYPSLPTSLPAVSQGFGQSTTATLGPSFD 480
Gv29.8          ALSYTSGHSPSPSASSGFSPQSRHSSTASSIMYPSLPTSLPAVSQGFGQSTTATLGPSFD 480
                ************************************************************

IMI             AGERRRYSGGMLQRARGAPPRSVEEPSGAVTPKASESTPPVGSPSSESSDVSDATREREE 540
Gv29.8          AGERRRYSGGMLQRARGAPPRSVEEPSGAVTPKASESTPPIGSPSSESSDVSDATREREE 540
                ****************************************:*******************

IMI             QYDRWVDNMRVIEALREYVQGRLKRGEFEQEYPSVNRIRKDVDAMDLEGKSRSPLANERP 600
Gv29.8          QYDRWVDNMRVIEALREYVQGRLKRGEFEQEYPSVNRIRKDVDAMDLEGKSRSPLANERP 600
                ************************************************************

IMI             MSKESNPLYPVLPVPGT 617
Gv29.8          MSKESNPLYPVLPVPGT 617
                *****************
```
