# Supplementary material for: PacC and pH–dependent transcriptome of the mycotrophic fungus Trichoderma virens
Source: BMC Genomics. 2013 Feb 28;14:138. doi: 10.1186/1471-2164-14-138 (PMC3618310; doi:10.1186/1471-2164-14-138)
Supplement: Additional file 3 — Verification of double-crossover events in transformants. This is a set of data confirming gene successful double crossover integration events. The linear map shows the pacC genomic region, with the relevant genes and markers indicated. Primer names are shown, with their locations and directions indicated by small arrows. PCR products are shown in the gel images; the source of the template DNA is shown in bold text below each lane, and the primer pairs used for the amplification are noted below the image, referring to the corresponding set of lanes. The primer names are also listed in the table (Expected sizes of PCR products from mutant validation), along with the predicted ampicon sizes. [file 1471-2164-14-138-S3.pdf]

Additional file 3 – Verification of double-crossover events in transformants.

The linear map shows the *pacC* genomic region, with the relevant genes and markers indicated. Primer names are shown, with their locations and directions indicated by small arrows. PCR products are shown in the gel images; the source of the template DNA is shown in bold text below each lane, and the primer pairs used for the amplification are noted below the image, referring to the corresponding set of lanes. The primer names are also listed in the table (Expected sizes of PCR products from mutant validation), along with the predicted ampicon sizes.

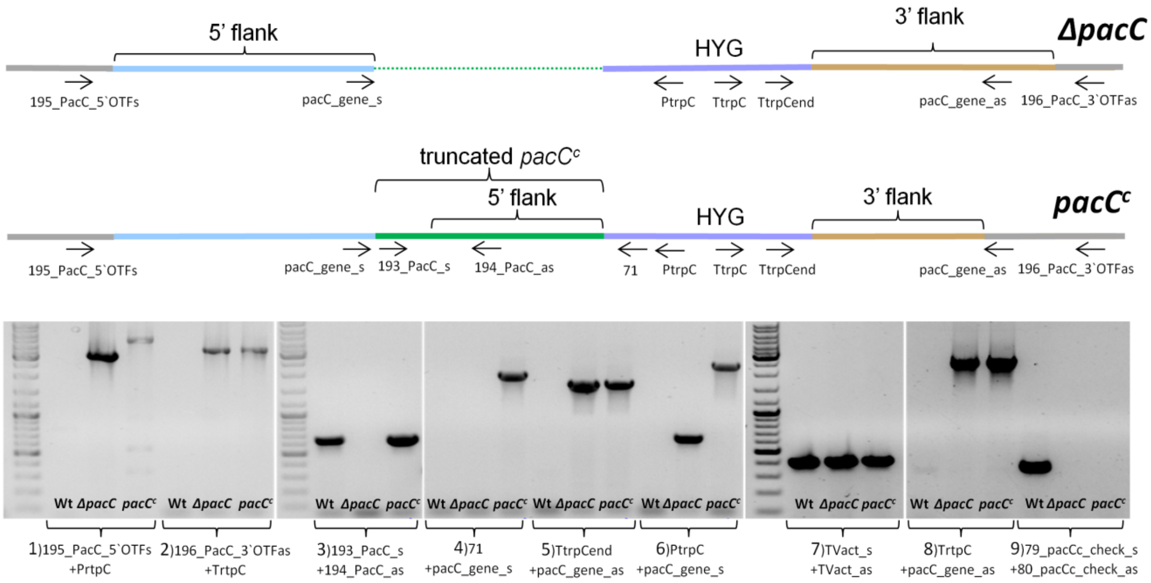

Expected sizes of PCR products from mutant validation

Primer pairs	Wt	ΔpacC	pacCc
1) 195_PacC_5`OTFs+PrtpC	-	3030 bp	4622 bp
2) 196_PacC_3`OTFas+TrtpC	-	3277 bp	3277 bp
3) 193_PacC_s+194_PacC_as	640 bp	-	640 bp
4) 71+pacC_gene_s	-	-	1758 bp
5) TtrpCend+pacC_gene_as	-	1592 bp	1592 bp

Primer pairs	Wt	ΔpacC	pacCc
6) PtrpC+pacC_gene_s	-	674 bp	2160 bp
7) TVact_s+TVact_as	395 bp	395 bp	395 bp
8) TrtpC+pacC_gene_as	-	2108 bp	2108 bp
9) 79_pacCc_check_s+80_pacCc_check_as	315 bp	-	-
