# Supplementary material for: PacC and pH–dependent transcriptome of the mycotrophic fungus Trichoderma virens
Source: BMC Genomics. 2013 Feb 28;14:138. doi: 10.1186/1471-2164-14-138 (PMC3618310; doi:10.1186/1471-2164-14-138)

Additional file 4- split-marker gene replacement strategy.

In the first round of PCR reactions, the flanking regions are amplified; in the second round, they are each fused to part of the hygromycin resistance cassette (HYG). For primer sequences see Additional File 14; the strategy and diagram are adapted from [46].

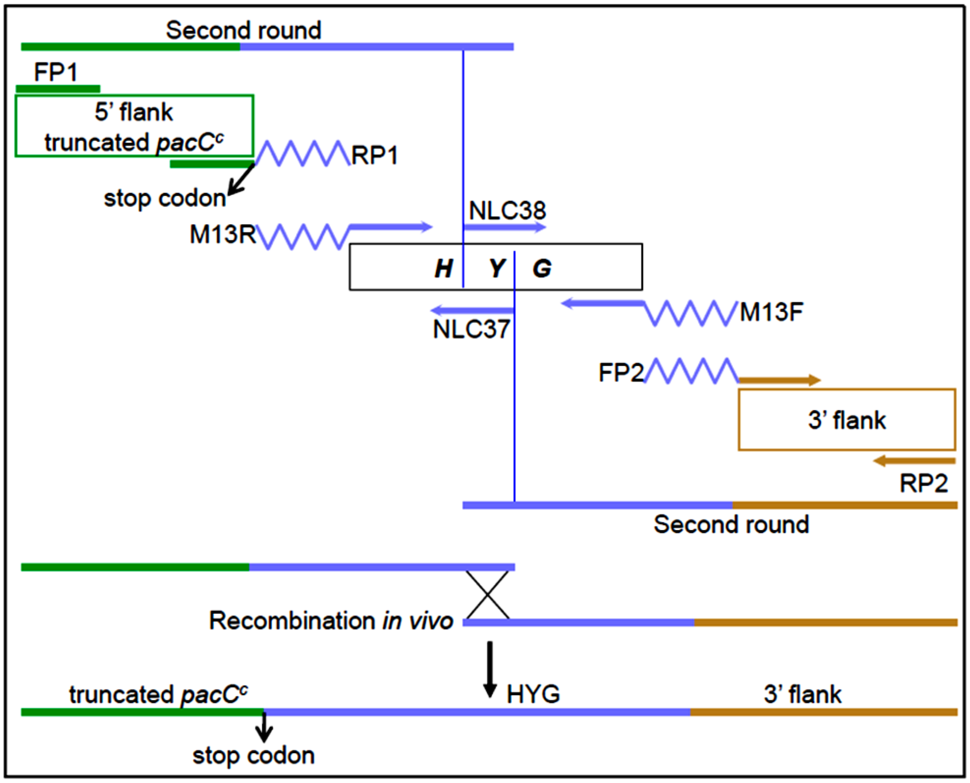

Supplement: Additional file 4 — Split-marker gene replacement strategy. This figure illustrates the split-marker strategy. In the first round of PCR reactions, the flanking regions are amplified; in the second round, they are each fused to part of the hygromycin resistance cassette (HYG). For primer sequences see Additional file 14; the strategy and diagram are adapted from [46]. [file 1471-2164-14-138-S4.pdf]
