# Supplementary material for: PacC and pH–dependent transcriptome of the mycotrophic fungus Trichoderma virens
Source: BMC Genomics. 2013 Feb 28;14:138. doi: 10.1186/1471-2164-14-138 (PMC3618310; doi:10.1186/1471-2164-14-138)

Additional file 5 – Growth rate of wild type and Δ*pacC*. The growth rate of the mutants relative to the wild type was determined by placing a 5-mm-diameter mycelial disk of the fungus in the center of a PDA plate and measuring the colony diameter at the indicated times. Values represent an average of 4 replicates. Error bars represent the standard deviation.

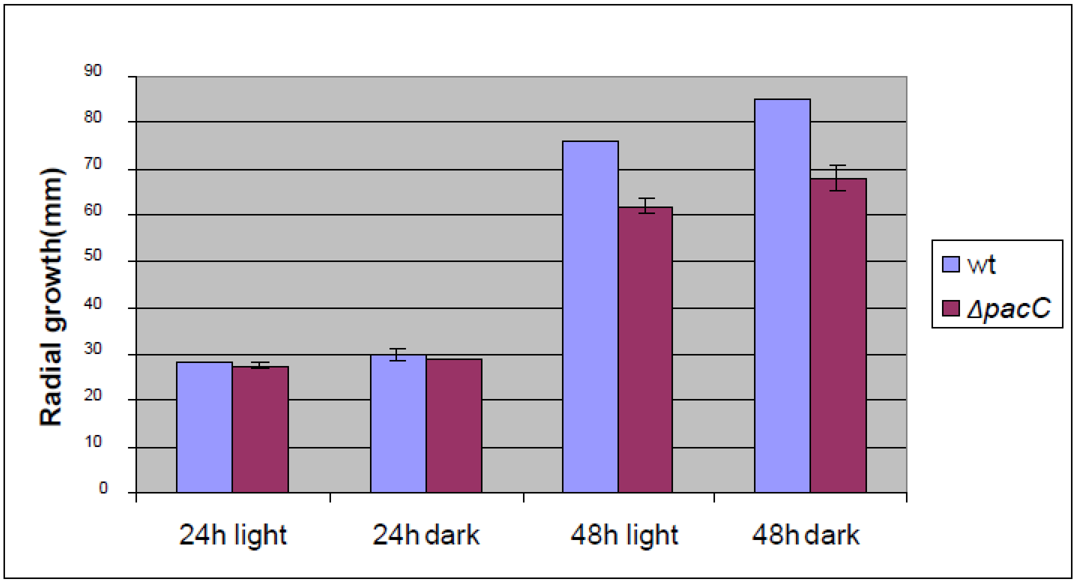

Supplement: Additional file 5 — Growth rate of wild type and ΔpacC. This graph shows growth rate of the ΔpacC strain relative to the wild type. Growth was measured by placing a 5-mm-diameter mycelial disk of the fungus in the center of a PDA plate and measuring the colony diameter at the indicated times. Values represent an average of 4 replicates. Error bars represent the standard deviation. [file 1471-2164-14-138-S5.pdf]
