# Supplementary material for: PacC and pH–dependent transcriptome of the mycotrophic fungus Trichoderma virens
Source: BMC Genomics. 2013 Feb 28;14:138. doi: 10.1186/1471-2164-14-138 (PMC3618310; doi:10.1186/1471-2164-14-138)

Additional file 6 - Morphology of wild type and Δ*pacC* mutant *T. virens*.

A 5-mm-diameter mycelial disk of the fungus was inoculated in the center of a PDA plate. The plates were incubated in the light and dark and colonies were photographed at the indicated times.

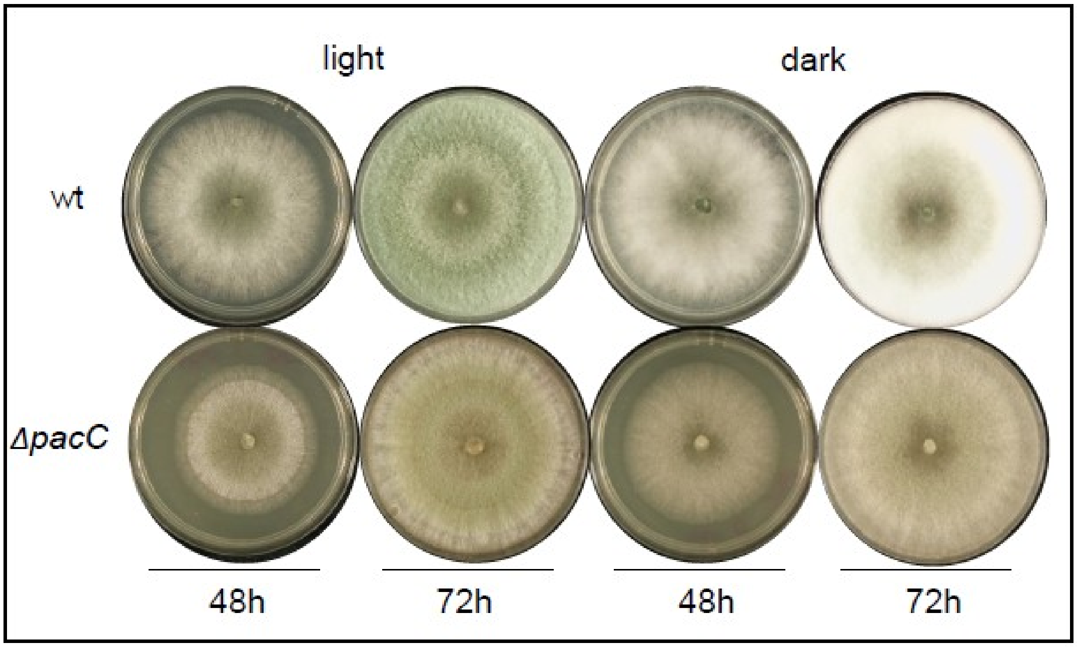

Supplement: Additional file 6 — Morphology of wild type and ΔpacC mutant T. virens. This figure is a photo of cultures, showing colony morphology. A 5-mm-diameter mycelial disk of the fungus was inoculated in the center of a PDA plate. The plates were incubated in the light and dark and colonies were photographed at the indicated times. [file 1471-2164-14-138-S6.pdf]
