# Supplementary material for: PacC and pH–dependent transcriptome of the mycotrophic fungus Trichoderma virens
Source: BMC Genomics. 2013 Feb 28;14:138. doi: 10.1186/1471-2164-14-138 (PMC3618310; doi:10.1186/1471-2164-14-138)
Supplement: Additional file 7 — Lists of differentially expressed genes from statistical analysis (Figure 5). List of protein ID numbers for each category of the genes shown in Figure 5 of the main text. [file 1471-2164-14-138-S7.pdf]

Additional file 7 - Lists of differentially expressed genes from statistical analysis (Figure 5).

The numbers indicate protein ID number according to v1.0 of the *T. virens* genome assembly (http://genome.jgi-psf.org/cgi-bin/searchGM?db=Trive1).

wt pH 8 > wt pH 4

62	23818	34210	44694	54160	63359	69787	79870
1583	23882	34248	44767	54245	64085	69800	79910
1598	24467	34285	44988	54471	64683	69813	80742
3912	26112	34473	45536	54659	64807	69821	80915
4594	27509	34514	45839	55670	64898	70125	80945
4916	28147	34827	46709	55713	64973	70206	81436
5073	28324	34872	46845	55793	65182	70300	81599
5637	28471	34908	46854	55818	65685	70450	81610
6309	28878	34950	46884	55945	65771	70657	81799
6352	29548	35100	46953	55997	66038	71019	82077
6898	29930	36320	47014	56058	66104	71022	82108
7366	30062	36493	47075	56118	66108	71023	82861
7406	30165	37069	47845	56294	66166	71270	83512
7723	30424	37111	47993	56547	66191	72203	84367
9444	30456	37740	49202	57290	66318	72350	84899
9562	30642	39063	49318	57481	66361	72478	85436
10799	30803	39685	49577	57790	66408	72547	85586
12255	31296	40391	49609	57864	66794	72673	86068
13146	31434	40784	49612	57880	66997	73234	86342
13221	31493	40829	49743	58008	67017	73257	86628
13611	31547	41088	49802	58111	67287	74348	86768
13820	31991	41593	49814	58166	67292	74393	87478
14412	32043	42016	50275	58169	67578	74715	87714
15079	32256	42239	50684	58256	67658	74722	88342
15416	32491	42564	51057	58340	67662	74915	88464
16120	32828	42568	51254	58634	67726	75374	88928
17086	32911	43455	51356	58793	67727	75759	89677
17565	32928	43564	51758	59177	67729	75793	89827
17916	32971	43674	51804	59860	67934	75976	91051
17934	33332	43724	51830	60268	68007	76182	91506
18141	33558	43815	51890	60434	68086	76487	91616
18327	33563	43838	51892	60578	68115	76620	91759
18378	33666	43901	51932	61062	68116	76718	92317
19142	33795	44039	51974	61173	68342	76948	92458
19266	33825	44141	53249	61203	68453	77023	92847
19331	33937	44194	53282	61853	68745	78116	110727
21063	34037	44199	53367	62353	69228	78136	110873
22482	34056	44273	53416	62483	69284	79195	110893
23185	34120	44392	53528	62839	69374	79277	111691
23200	34162	44490	54060	62940	69456	79497	111897
							111979

wt pH 8 > wt pH 4 and wt pH 8 > *ΔpacC* pH 8

62	28324	34827	51804	58008	66794	74348	80915
1583	28471	34950	51974	58111	67287	74915	85436
5073	28878	37069	53416	58340	67662	75759	85586
5637	31296	39685	54160	60268	68086	76487	86342

6898	32256	40391	54471	61173	69284	76620	88464
10799	32491	42239	55713	61853	69374	76948	88928
13820	33666	42568	55793	62353	70125	77023	89677
16120	33937	43815	55945	62940	71270	78136	91616
18327	34037	44039	55997	64085	72203	79497	111897
21063	34210	47075	56118	65182	72350	79870	
27509	34248	47845	56294	66038	72673	79910	
28147	34514	49577	57790	66108	73257	80742	

wt pH 8 > wt pH 4 and wt pH 8 > *ΔpacC* pH 8 and
ΔpacC pH 8 = *ΔpacC* pH 4 and wt pH 4 ≥ *ΔpacC* pH 4

62	28324	34514	47845	57790	66108	73257	86342
1583	28471	34827	51974	58008	67287	74348	88464
5073	28878	34950	53416	58111	67662	75759	89677
5637	31296	37069	54160	58340	68086	76487	111897
6898	32256	39685	54471	60268	69284	76620	
10799	32491	40391	55713	61173	69374	76948	
13820	33666	42239	55945	61853	70125	77023	
21063	33937	42568	55997	62353	71270	78136	
27509	34037	43815	56118	64085	72203	79870	
28147	34248	47075	56294	65182	72673	80915	

wt pH 8 > wt pH 4 and wt pH 8 > *ΔpacC* pH 8 and *ΔpacC* pH 8 > *ΔpacC* pH 4

16120	49577	55793	66038	72350	79497	85436	91616
18327	51804	62940	66794	74915	79910	88928	

wt pH 8 > wt pH 4 and wt pH 8 > *ΔpacC* pH 8 and wt pH 4 > *ΔpacC* pH 4

18327	33666	33937	40391	66108	74915	79497	85436

wt pH 8 < wt pH 4

49	31415	44387	52534	61140	69509	78714	86314
1402	31473	44709	52801	61437	69626	78735	86397
1900	31655	45164	52839	61596	69842	78759	86792
2224	31892	45299	52876	61788	70229	79145	86828
3262	32650	45391	52981	61957	70389	79303	87170
4165	32837	45407	53225	62266	70542	79337	87711
4561	32844	45560	53228	62289	71204	79354	87721
4744	32952	45768	53370	62497	71429	79625	88246
5211	32969	45779	53581	62502	71577	79679	88285
6421	33397	45894	53582	62590	71692	79681	88374
8279	33658	46143	53730	62656	71997	79823	88538
8451	33927	46411	54100	62665	72390	80215	88881
8690	34363	46453	54531	62683	72446	80284	88883
10390	34822	46500	54747	62760	72458	80526	89165
12616	34877	46596	54997	62870	72860	80583	89424
13148	34881	46639	55211	63140	73176	80591	89738
13236	35984	46824	55675	63496	73483	80929	89740
13994	36112	46990	55747	63835	73504	81170	89941
14736	36187	47116	55803	63940	73591	81561	91002
14863	36701	47120	55809	64335	73622	81710	91256
15214	37118	47152	55825	64534	73671	81735	91258
15665	37142	47770	56771	64838	73922	81832	91753
16988	37216	47789	57212	64931	74289	81869	92044
17064	37560	47998	57354	65142	74291	81957	92048

18652	38104	48079	57476	65382	74397	82134	92112
19422	38198	48261	57871	65436	74792	82254	92435
21187	38237	48610	58016	65978	74836	82486	92614
22298	38517	48794	58191	65980	75173	82546	92648
23422	39626	49873	58221	66237	75241	82626	92958
26720	39703	49894	58255	66295	75395	82648	110548
27962	39763	49896	58320	66558	75728	82975	110616
28241	40073	50212	58356	67765	75806	83020	110789
28425	40076	50413	58495	67871	76008	83056	110799
28884	40373	50552	58538	68002	76647	83099	111175
29072	40635	50578	58567	68122	76953	83793	111364
29306	41159	50666	58597	68271	77102	84263	111431
29650	41165	50865	58728	69068	77460	84449	111460
29667	41304	50949	59067	69133	77465	84563	111469
29738	41337	51050	59188	69135	77579	84951	111830
29941	43330	51083	59343	69222	77829	85675	111832
30396	43385	51305	59538	69343	78031	85743	111853
30786	43673	51849	60797	69429	78069	86235	111957
31390	44127	52018	60819	69432	78413	86266	111966

wt pH 8 < wt pH 4 and wt pH 8 < Δ*pacC* pH 8

1900	31415	45164	53370	58255	68271	78759	86792
3262	34822	45768	53581	58495	69429	79145	88246
13148	34877	46411	53582	59538	69432	81869	88285
22298	36701	47770	53730	62266	71577	81957	89738
23422	37142	48610	55803	62665	72390	82648	89740
28425	40076	49894	55825	62760	72458	83793	92435
28884	40635	51083	58016	63496	75173	85675	111431
29072	44387	52534	58191	64931	77460	85743	111460

wt pH 8 < wt pH 4 and wt pH 8 < Δ*pacC* pH 8 and
Δ*pacC* pH 8 = Δ*pacC* pH 4 and wt pH 4 ≤ Δ*pacC* pH 4

3262	34877	46411	53370	59538	69429	81869	89738
22298	36701	47770	55803	62266	69432	82648	89740
23422	40076	48610	55825	62665	72458	83793	111431
28425	40635	49894	58016	63496	75173	85675	111460
28884	45164	51083	58191	64931	77460	86792	
31415	45768	52534	58255	68271	78759	88285	

wt pH 8 < wt pH 4 and wt pH 8 < Δ*pacC* pH 8 and Δ*pacC* pH 8 < Δ*pacC* pH 4

13148	37142	53581	53730	62760	72390	81957	88246
34822	44387	53582	58495	71577	79145	85743	92435

wt pH 8 < wt pH 4 and wt pH 8 < Δ*pacC* pH 8 and wt pH 4 < Δ*pacC* pH 4

13148	37142	52534	53730	78759	85675	89740
34822	48610	53581	69432	79145	85743	92435
36701	49894	53582	72458	82648	88246	
