# Supplementary material for: PacC and pH–dependent transcriptome of the mycotrophic fungus Trichoderma virens
Source: BMC Genomics. 2013 Feb 28;14:138. doi: 10.1186/1471-2164-14-138 (PMC3618310; doi:10.1186/1471-2164-14-138)
Supplement: Additional file 8 — Expression profiles of PacC-dependent, alkaline pH upregulated genes, and available annotation. Details of regulated genes are given here. Secreted proteins are indicated by a + if predicted at a SignalP score of at least 0.7. The number of predicted PacC binding sites (GCCARG, see text) in the 1 kb region upstream of the start codon is given in the last column. Expression profiles (fold expression at pH 8 compared to pH 4, and wt compared to ΔpacC at pH 8) are given. Annotation by homology: entries are best hits by BLAST, E values are given in the following column. [file 1471-2164-14-138-S8.pdf]

Additional file 9 - Expression profiles of PacC-dependent, alkaline pH upregulated genes, and available annotation. Secreted proteins are indicated by a + if predicted at a SignalP score of at least 0.7. The number of predicted PacC binding sites (GCCARG, see text) in the 1 kb region upstream of the start codon is given in the last column. Expression profiles (fold expression at pH 8 compared to pH 4, and wt compared to Δ*pacC* at pH 8) are given. Annotation by homology: entries are best hits by BLAST, E values are given in the following column.

Protein ID	SignalP	Annotation by homology	E value	Fold wt8/wt4	Fold wt8/Δ*pacC*8	number of binding sites
1583		fungal specific transcription factor [*Colletotrichum higginsianum*]	0	5	3	3
5073		hypothetical protein		15	78	2
5637		hypothetical protein		4	4	2
6898		mannosylphosphate transferase [*Grosmannia clavigera* kw1407]	4.00E-98	16	37	4
10799	+	putative Na+/H+ antiporter CNH1 [*Metarhizium anisopliae* ARSEF 23]	0	9	9	2
13820		eukaryotic aspartyl protease [*Glomerella graminicola* M1.001]	4.00E-142	5	2	5
16120				4	2	1
18327		ribonuclease Trv [*Verticillium albo-atrum* VaMs.102]	6.00E-126	63	48	3
21063	+	DNA repair protein rad5 [*Pyrenophora tritici-repentis* Pt-1C-BFP]	2.00E-66	10	7	1
27509		cyanovirin-N [*Aspergillus niger* CBS 513.88]	9.00E-08	14	24	4
28147		hypothetical protein		2.2E+04	4.6E+04	3
28324		related to DNA damage-responsive protein 48 [*Piriformospora indica* DSM 11827]	1.00E-29	8	3	5
28471		glycoside hydrolase family 76 protein [*Trichoderma virens* Gv29-8]	0	3	3	3
28878		alcohol dehydrogenase [*Aspergillus oryzae* RIB40]	2.00E-88	4	4	3
31296		DNA/RNA helicase [*Grosmannia clavigera* kw1407]	7.00E-67	4	6	2
32256		MFS transporter [*Metarhizium acridum* CQMa 102]	0	8	4	3
32491	+	glutathione-dependent formaldehyde-activating enzyme [Glomerella graminicola M1.001]	2.00E-51	3	7	2
33666	+	GDP-fucose transporter-like protein [*Trichoderma atroviride* IMI 206040]	7.00E-154	31	6.8E+02	2
33937	+	pyruvyltransferase [*Schizosaccharomyces japonicus* yFS275]	1.00E-44	24	58	0
34037		similar to MFS multidrug transporter [*Botryotinia fuckeliana*]	1.00E-111	5	2	1
34210				9	2	2
34248		betaine aldehyde dehydrogenase [*Metarhizium anisopliae* ARSEF 23]	0	58	3	2
34514		C4-dicarboxylate transporter/malic acid transporter [*Glomerella graminicola* M1.001]	0	47	47	6
34827		cation transporting ATPase [*Trichoderma virens* Gv29-8]	0	2.0E+03	8.3E+02	8
34950		hypothetical protein		6.7E+03	1.3E+07	0
37069		GNAT family acetyltransferase, putative [*Metarhizium anisopliae* ARSEF 23]	3.00E-133	6	2	3

39685		SPFH domain/Band 7 family protein [*Glomerella graminicola* M1.001]	0	8	2	2
40391		transcription factor PacC [*Hypocrea lixii*]	0	27	3.5E+08	9
42239	+	calcineurin-like phosphoesterase, putative [*Metarhizium acridum* CQMa 102]	0	3	3	3
42568		glutathione S-transferase [*Neurospora tetrasperma* FGSC 2509]	3.00E-07	7	5	3
43815		glycosyltransferase [*Trichoderma reesei* QM6a]	0	2	2	2
44039				10	2	3
47075		arginine deiminase type-3 [*Metarhizium anisopliae* ARSEF 23]	0	1.2E+02	22	2
47845	+	srpk, putative [*Metarhizium anisopliae* ARSEF 23]	3.00E-49	20	9	6
49577	+	hypothetical protein		80	6	2
51804				23	3	4
51974	+	hypothetical protein		5	6.5E+06	4
53416		hypothetical protein		38	12	3
54160	+	ribonuclease T2 precursor [*Cordyceps militaris* CM01]	7.00E-171	2	2	2
54471		Thioredoxin-(TRX) like protein [*Cupriavidus metallidurans* CH34]	9.00E-54	5	18	2
55713		HpcH/HpaI aldolase/citrate lyase family protein [*Colletotrichum higginsianum*]	6.00E-102	6	4	1
55793	+			3	2	3
55945		SNARE-dependent exocytosis protein (Sro7), putative [*Cordyceps militaris* CM01]	0	3	4	2
55997	+	hypothetical protein		8	3	3
56118		sodium/hydrogen exchanger family protein [*Colletotrichum higginsianum*]	5.00E-164	3	3	1
56294		PX domain protein [*Metarhizium anisopliae* ARSEF 23]	0	12	4	1
57790		queuine tRNA-ribosyltransferase-like protein [*Chaetomium thermophilum*]	9.00E-05	3	3	4
58008		tagatose-bisphosphate aldolase [*Trichoderma reesei* QM6a]	4.00E-73	2.1E+03	4.8E+03	2
58111	+	glycoside hydrolase family 76 protein [*Trichoderma virens* Gv29-8]	0	3.2E+02	4.6E+04	6
58340		hypothetical protein		3	4	1
60268	+	similar to glutathione-dependent formaldehyde-activating gfa [*Botryotinia fuckeliana*]	3.00E-13	69	25	1
61173		AAA family ATPase, putative [*Cordyceps militaris* CM01]	2.00E-30	4	4	3
61853	+	heterokaryon incompatibility protein Het-C [*Glomerella graminicola* M1.001]	0	2	5	6
62353		hypothetical protein		5	5	1
62940	+	WSC domain protein [*Neosartorya fischeri* NRRL 181]	2.00E-62	4.3E+02	24	2
64085	+	hypothetical protein		3	4	2
65182		hypothetical protein		5	5	0
66038	+			8	2	3
66108	+	hypothetical protein		3	12	2
66794	+			4	2	2
67287		NADP(+)-dependent dehydrogenase, putative [*Aspergillus clavatus* NRRL 1]	1.00E-52	6	2	2
67662		cation transporting ATPase [*Trichoderma virens* Gv29-8]	0	4.1E+05	3.4E+07	3
68086	+	related to ZRT2-Zinc transporter II [*Sporisorium reilianum* SRZ2]	8.1	2.2E+03	6.7E+03	2

69284		cation transporting ATPase [*Trichoderma virens* Gv29-8]	0	3	2	6
69374	+	NACHT and ankyrin domain protein [*Trichoderma virens* Gv29-8]	0	2	2	1
70125		hypothetical protein		7	48	1
71270	+	secreted aspartic proteinase [*Hypocrea lixii*]	0	5	2	3
72203		fructose-bisphosphatase [*Trichoderma reesei* QM6a]	0	2	2	2
72350				3	2	5
72673		putative Plasma membrane proteolipid 3 [*Glarea lozoyensis* 74030]	7.00E-31	1	2	6
73257	+	copper radical oxidase [*Colletotrichum higginsianum*]	0	2	2	2
74348	+	putative glyoxal oxidase precursor [*Metarhizium anisopliae* ARSEF 23]	0	5	2	7
74915	+	plasma membrane ATPase [*Trichoderma atroviride* IMI 206040]	0	5	4	8
75759	+	pH-response regulator protein palC [*Metarhizium acridum* CQMa 102]	0	12	1.8E+02	4
76487		C2 domain containing protein [*Metarhizium anisopliae* ARSEF 23]	0	5	3	7
76620	+	endonuclease/exonuclease/phosphatase family protein [*Metarhizium acridum* CQMa 102]	0	42	3.2E+05	3
76948	+	5'-nucleotidase precursor [*Metarhizium anisopliae* ARSEF 23]	0	5	7	2
77023	+	methyltransferase [*Arthroderma otae* CBS 113480]	2.00E-114	3.9E+03	3.2E+05	3
78136		GNAT family acetyltransferase, putative [*Metarhizium anisopliae* ARSEF 23]	4.00E-41	3	2	1
79497	+	Oligopeptide transporter [*Cordyceps militaris* CM01]	0	8	5	6
79870		vacuolar import and degradation protein [*Verticillium dahliae* VdLs.17]	0	2	2	3
79910		carbohydrate-binding module family 50 protein [*Trichoderma virens* Gv29-8]	6.00E-36	8	2	1
80742				3	3	4
80915	+	sodium/phosphate symporter, putative [*Metarhizium acridum* CQMa 102]	0	1.4E+02	2.7E+02	2
85436		dicarboxylic amino acid permease [*Metarhizium acridum* CQMa 102]	0	5	3	2
85586				11	3	3
86342	+	glycoside hydrolase family 47 protein [*Trichoderma virens* Gv29-8]	0	3	3	4
88464		integral membrane protein [*Aspergillus kawachii* IFO 4308]	3.00E-70	3	2	2
88928	+	putative aspartic endopeptidase [*Hypocrea lixii*]	0	7	3	2
89677	+	ZIP Zinc transporter [*Glomerella graminicola* M1.001]	2.00E-145	2.6E+02	34	8
91616	+	transaldolase/ glucose-6-phosphate isomerase [*Bradyrhizobiaceae bacterium* SG-6C]	2.7	1.1E+02	14	3
111897	+	Protein kinase-like domain [*Cordyceps militaris* CM01]	3.00E-65	20	34	2
