# Supplementary material for: PacC and pH–dependent transcriptome of the mycotrophic fungus Trichoderma virens
Source: BMC Genomics. 2013 Feb 28;14:138. doi: 10.1186/1471-2164-14-138 (PMC3618310; doi:10.1186/1471-2164-14-138)

**Additional file 8 –Mutants in P type ATPase *ena1* are similar to wt in plate assays for growth and confrontation.** The strains were inoculated on PDA plates photographed at 11 days from above to show overgrowth and sporulation (A) or below to show pH indicator color (B). The wt and *Δena1* strains were grown in confrontation with two plant pathogens.

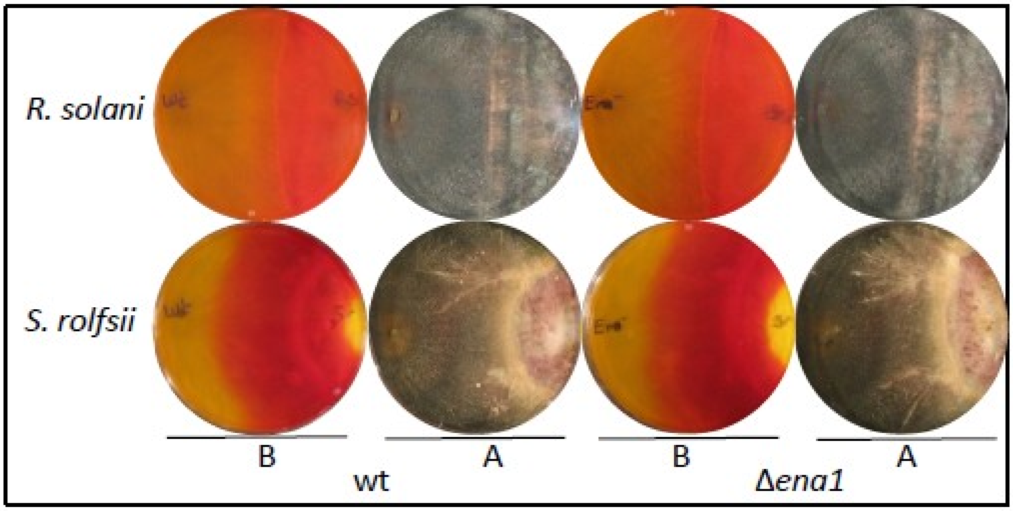

Supplement: Additional file 9 — Mutants in P type ATPase Δena1 are similar to wt in plate assays for growth and confrontation. Photos of cultures grown on PDA plates, taken at 11 days from above to show overgrowth and sporulation (A), or below to show pH indicator color (B). The wt and Δena1 strains were grown in confrontation with two plant pathogens. [file 1471-2164-14-138-S9.pdf]
