# Supplementary material for: PacC and pH–dependent transcriptome of the mycotrophic fungus Trichoderma virens
Source: BMC Genomics. 2013 Feb 28;14:138. doi: 10.1186/1471-2164-14-138 (PMC3618310; doi:10.1186/1471-2164-14-138)
Supplement: Additional file 10 — Expression profiles of PacC-dependent, acid pH upregulated genes, and available annotation. Details of regulated genes are given here. Secreted proteins are indicated by a + if predicted at a SignalP score of at least 0.7. The number of predicted PacC binding sites (GCCARG, see text) in the 1 kb region upstream of the start codon is given in the last column. Expression profiles (fold expression at pH 8 compared to pH 4, and wt compared to ΔpacC at pH 8) are given. Annotation by homology: entries are best hits by BLAST, E values are given in the following column. [file 1471-2164-14-138-S10.pdf]

Additional file 10 - Expression profiles of PacC-dependent, acid pH upregulated genes, and available annotation. Secreted proteins are indicated by a + if predicted at a SignalP score of at least 0.7. The number of predicted PacC binding sites (GCCARG, see text) in the 1 kb region upstream of the start codon is given in the last column. Expression profiles (fold expression at pH 8 compared to pH 4, and wt compared to Δ*pacC* at pH 8) are given. Annotation by homology: entries are best hits by BLAST, E values are given in the following column.

TargetID	SignalP	Annotation by homology	E value	Fold Wt4/Wt8	Fold *pacC*8/Wt8	number of binding sites
1900	+			4	3	5
3262	+	similar to purine-cytosine permease [*Botryotinia fuckeliana*]	0	3	3	0
13148	+			7	15	1
22298		glycosyltransferase family 31 protein [*Trichoderma virens* Gv29-8]	0	16	39	2
23422	+	putative P450 monooxygenase [*Mycosphaerella graminicola* IPO323]	1E-108	4	6	0
28425		putative family C-likeG-protein-coupled receptor [*Trichoderma atroviride* IMI 206040]	5E-171	4	3	0
28884	+	predicted small secreted cysteine-rich protein	1E-118	5	9	1
29072	+			7	21	2
31415		NADH:flavin oxidoreductase/NADH oxidase [*Trichoderma atroviride* IMI 206040]	0	3	4	1
34822				7	9	1
34877		acyltransferase, putative [*Metarhizium anisopliae* ARSEF 23]	0	10	10	0
36701	+	aldehyde dehydrogenase [*Aspergillus kawachii* IFO 4308]	1E-145	2	3	0
37142				16	42	3
40076				5	7	3
40635	+	hydrophobin, partial [*Trichoderma virens* Gv29-8]	1E-70	3	7	1
44387				5	3	4
45164		short-chain dehydrogenase [*Colletotrichum higginsianum*]	9E-94	3	3	0
45768		cytosine-purine permease [*Trichoderma reesei* QM6a]	0	5	6	1
46411		major facilitator superfamily transporter [*Glomerella graminicola* M1.001]	0	3	5	0
47770	+	Na+/proline symporter [*Trichoderma reesei* QM6a]	0	5	5	2
48610		major facilitator superfamily transporter [*Glomerella graminicola* M1.001]	0	3	5	3
49894		monooxygenase, putative [*Aspergillus flavus* NRRL3357]	2E-101	2	3	4
51083		ctr copper transporter family protein [*Grosmannia clavigera* kw1407]	1E-19	23	1.34E+02	1
52534		IQ calmodulin-binding motif protein [*Metarhizium acridum* CQMa 102]	0	1.87E+02	3.83E+02	1
53370		cytochrome P450, putative [*Metarhizium anisopliae* ARSEF 23]	2E-163	2	2	1
53581				3	4	0

53582				3	3	0
53730				5	5	1
55803		fatty acid oxygenase, putative [*Metarhizium acridum* CQMa 102]	0	3	5	1
55825	+	cytochrome P450 monooxygenase [*Metarhizium acridum* CQMa 102]	0	3	3	2
58016	+	MFS (Major Facilitator Superfamily) toxin efflux pump (AflT) [*Metarhizium acridum* CQMa 102]	0	2	2	2
58191		peptide transporter [*Trichoderma reesei* QM6a]	0	3	9	0
58255		adenosine deaminase [*Glomerella graminicola* M1.001]	0	3	5	0
58495				5	4	1
59538		short-chain dehydrogenase [*Colletotrichum higginsianum*]	7E-97	8	11	1
62266		LPS-induced tumor necrosis factor alpha factor [*Cordyceps militaris* CM01]	2E-06	5	11	6
62665		bifunctional catalase/peroxidase [*Trichoderma reesei* QM6a]	0	7	11	3
62760				3.95E+02	1.04E+02	2
63496	+	triose-phosphate transporter [*Glomerella graminicola* M1.001]	1E-142	2	3	5
64931		pleiotropic drug resistance protein [*Trichoderma atroviride* IMI 206040]	0	4	3	1
68271	+	N-acetylglucosaminyl-phosphatidylinositol de-N-acetylase, a glycan bosynthesis protein [*Trichoderma virens* Gv29-8]	0	3	5	3
69429		similar to ferric reductase like transmembrane component [*Botryotinia fuckeliana*]	2E-143	3	3	0
69432		transmembrane amino acid transporter protein [*Trichoderma reesei* QM6a]	0	30	36	1
71577				4	4	2
72390				4	4	1
72458		multidrug resistance-associated protein [*Trichoderma atroviride* IMI 206040]	0	3	3	1
75173	+	gaba permease [*Trichoderma reesei* QM6a]	0	2	3	1
77460	+	hypothetical protein		4	4	0
78759		major facilitator superfamily transporter [*Glomerella graminicola* M1.001]	0	4	8	2
79145				4	5	1
81869	+	chitinase 3 [*Coccidioides posadasii*]	4E-14	5	6	2
81957	+	xenobiotic compound monooxygenase [*Magnaporthe oryzae* 70-15]	0	9	7	1
82648	+	tripeptidyl peptidase A [*Neosartorya fischeri* NRRL 181]	0	18	3.76E+02	1
83793		ABC-transporter [*Metarhizium anisopliae* ARSEF 23]	0	3	2	1
85675		transmembrane amino acid transporter [*Trichoderma reesei* QM6a]	0	5	27	3
85743		sulfite oxidase [*Trichoderma reesei* QM6a]	0	4	3	1
86792		cytochrome P450 [*Glomerella graminicola* M1.001].	0	9	9	1
88246		bleomycin hydrolase, putative [*Metarhizium anisopliae* ARSEF 23]	0	3	3	1
88285		hydrolase, alpha/beta hydrolase fold family [*Metarhizium anisopliae* ARSEF 23]	6E-88	7	9	2
89738	+	Glycosyltransferase Family 32 protein	0	8	26	2
89740	+	Glycosyltransferase Family 32 protein	0	6	18	3
92435		alcohol dehydrogenase [*Cordyceps militaris* CM01	6E-180	5	8	0
111431	+	hypothetical protein		2	3	1
111460		hypothetical protein		3	2	0
