# Supplementary material for: PacC and pH–dependent transcriptome of the mycotrophic fungus Trichoderma virens
Source: BMC Genomics. 2013 Feb 28;14:138. doi: 10.1186/1471-2164-14-138 (PMC3618310; doi:10.1186/1471-2164-14-138)

Additional file 12 - Disruption of *pacC* by homologous integration.

The diagram shows the scheme used for replacement of the entire *pacC* coding sequence. HYG indicates selectable marker cassette (for full details, see Methods, main text).

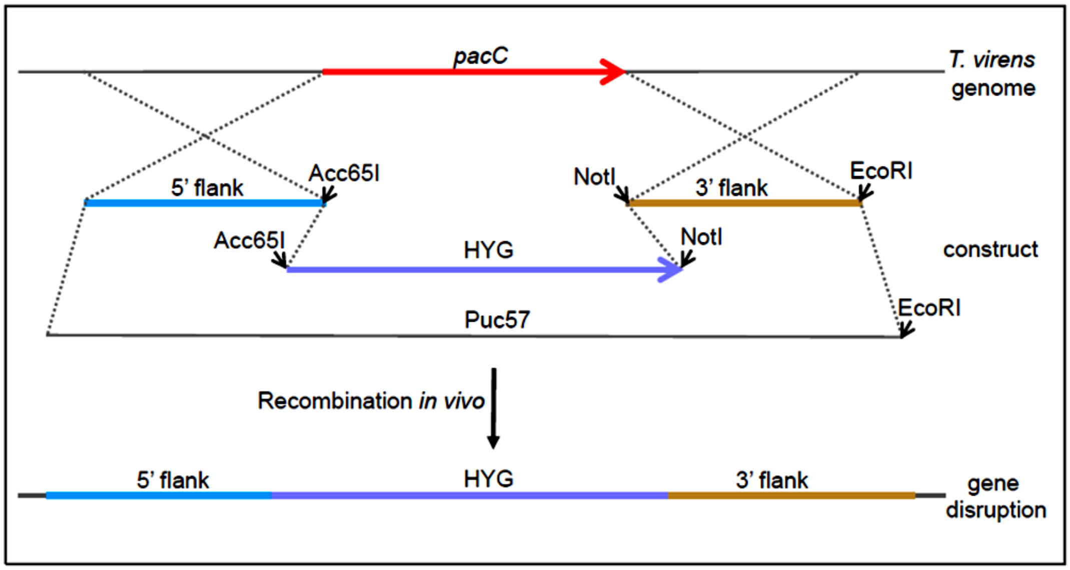

Supplement: Additional file 11 — Disruption of pacC by homologous integration. The diagram shows the scheme used for replacement of the entire pacC coding sequence. HYG indicates selectable marker cassette (for full details, see Methods, main text). [file 1471-2164-14-138-S11.pdf]
