# Supplementary material for: PacC and pH–dependent transcriptome of the mycotrophic fungus Trichoderma virens
Source: BMC Genomics. 2013 Feb 28;14:138. doi: 10.1186/1471-2164-14-138 (PMC3618310; doi:10.1186/1471-2164-14-138)
Supplement: Additional file 12 — Primers for gene knock-out. Sequences and names of the primers used for knock-out of pacC and confirmation of the integration event are given. [file 1471-2164-14-138-S12.pdf]

## Additional file 13 - Primers for gene knock-out

Sequences and names of the primers used for knock-out of *pacC* and confirmation of the integration event are given.

| Pairs | Primer | Sequence |
|---|---|---|
| ***ΔpacC*** | | |
| **Pair 1** | PacC 3’ flank as | CCATGTATTATTGAATTCAAACTCGG |
| | PacC 3’ flank s | AAGTCCAGGCGGCCGCTTGC |
| **Pair 2** | PacC 5’ flank as | GTACCCATCCGTCATCACTTACAT |
| | PacC 5’ flank s | TGCATATTCGCCAGCTCTATACG |
| ***pacC* deletion confirmation** | 195_PacC_5`OTFs | TCCTCCCTAGTCCATGGTATCGG |
| | 196_PacC_3`OTFas | GCTCCATCGATGTCAACACTGTG |
| | 193_PacC_s | CGCCTCACCTACAGATGCTTCG |
| | 194_PacC_as | GCTTGTGGTCTGTAGTTCATGCCG |
