# Supplementary material for: PacC and pH–dependent transcriptome of the mycotrophic fungus Trichoderma virens
Source: BMC Genomics. 2013 Feb 28;14:138. doi: 10.1186/1471-2164-14-138 (PMC3618310; doi:10.1186/1471-2164-14-138)
Supplement: Additional file 13 — Primers for split-marker gene replacement strategy. This is a primer list for split-marker gene replacement. In the Table, lower case indicates sequences that are not complementary to the template in the first PCR step. In the second PCR step, these sequences, which are complementary to the ends of the selectable marker, allow joining of the fragments. [file 1471-2164-14-138-S13.pdf]

Additional file 14 - Primers for split-marker gene replacement strategy.

Lower case indicates sequences that are not complementary to the template in the first PCR step. In the second PCR step, these sequences, which are complementary to the ends of the selectable marker, allow joining of the fragments.

Pairs	Primer	Sequence
pacC^c		
**Pair 1**	FP1PacC^c	CAACCCGGCATGAACTACAG
	RP1PacC^c	tcctgtgtgaaattgttatccgctTTAGACAGATCGTGGCGGTGCA
**Pair 2**	FP2PacC^c	gtcgtgactgggaaaacGATGTTTGTTGTATGAGGTGGC
	RP2PacC^c	GGGAAGACCGAAAATGAAGAG
**Pair 3**	M13Rhyg	AGCGGATAACAATTTCACACAGGA
	NLC37	GGATGCCTCCGCTCGAAGTA
**Pair 4**	M13Fhyg	CGCCAGGGTTTTCCCAGTCACGAC
	NLC38	CGTTGCAAGACCTGCCTGAA
**Pair 5**	71	GCGCTTGGCGTAATCATGGTC
	pacC_gene_s	GACTTTTCTGCTGCCCCACCACGAC
**Pair 6**	TtrpCend`	GTGAATGCTCCGTAACACCCAATAC
	pacC_gene_as	CGAGACGGCGGCAAACGAAAGCG
	79_pacCc_check_s	AAGGCAAGCGAATCCACTCC
	80_pacCc_check_as	GTACCAGGAACAGGCAGGACG
